# New Insecticidal Agents from Halogenation/Acylation of the Furyl-Ring of Fraxinellone

**DOI:** 10.1038/srep35321

**Published:** 2016-10-24

**Authors:** Yong Guo, Ruige Yang, Hui Xu

**Affiliations:** 1 Research Institute of Pesticidal Design & Synthesis, College of Sciences/Plant Protection, Northwest A&F University, Yangling 712100, Shaanxi Province, P. R. China; 2 School of Pharmaceutical Sciences, Zhengzhou University, Zhengzhou 450001, Henan Province, P. R. China

## Abstract

Introduction of the halogen atom or the acyl group at the C-ring of fraxinellone was investigated. Some unexpected halogenation products were obtained with the different chlorination/bromination reagents, and their possible reaction mechanisms were also proposed. Seven key steric structures of 2a’, 2b, 2b’, 2c’, 3a, 3b, and one isomer (5’α-Cl) of 2a were further confirmed by single-crystal X-ray diffraction. Especially compounds 2a, 2a’, 3a and 3c exhibited more potent insecticidal activity than toosendanin. Some structure-activity relationships of tested compounds were also described.

*Mythimna separata* Walker is a typical lepidopteran pest widely distributed in the world, and its intermittent outbreaks could lead to complete crop loss^1^. Although synthetic agrochemicals are used to control insect pests, the increasing application has resulted in resistance in pests resistance problems, ecological disturbances, and environmental problems^2^,^3^. Therefore, search of the new potential alternatives to effectively and selectively control insect pests is highly desirable in the agricultural field^4^,^5^,^6^,^7^,^8^.

Fraxinellone (**1**, Fig. 1) is isolated as a degraded limonoid from many Meliaceae and Rutaceae plants, and exhibits a variety of interesting properties, including the antiinflammatory bowel disease^9^, neuroprotective^10^, and insecticidal activities^11^,^12^. In our previous reports, compound **1** was modified at its A-ring (C-4/C-10 position) and B-ring (C-1/C-8 position), respectively. We found that some compounds of fraxinellone-based esters^13^,^14^ (**I**, **II**, and **V-VII**, Fig. 1) and hydrazones^15^ (**III** and **IV**, Fig. 1) displayed higher insecticidal activity than toosendanin against pre-third-instar larvae of *Mythimna separata*. The preliminary structure-activity relationships indicated that the lactone (B-ring) of compound **1** was vital for the insecticidal activity; the double bond at the C-2 position of compound **1** was not necessary for the insecticidal activity; conversion of the oxygen atom of carbonyl group on the lactone of compound **1** to a sulfur one decreased the insecticidal activity. However, up to now the influence of the C-ring (furyl ring) of compound **1** to its insecticidal activity was not clear. As part of our ongoing search for new natural-product-based insecticidal agents^16^,^17^,^18^, herein we wanted to prepare a series of new fraxinellone derivatives (**VIII**, Fig. 1) by introducing the halogen atom or the acyl group at the C-ring of **1** as insecticidal agents.

## Methods

### Materials and Instruments

All chemical reagents were purchased and utilized without further purification. Solvents were used directly or treated with standard methods before use. Melting points (mp) were determined on a XT-4 digital melting point apparatus (Beijing Tech Instrument Co., Ltd., Beijing, China) and were uncorrected. Infrared spectra (IR) were recorded on a Bruker TENSOR 27 spectrometer. Optical rotation was measured on a Rudolph Research Analytical Autopol III automatic polarimeter. Proton nuclear magnetic resonance spectra (^1^H NMR) and carbon nuclear magnetic resonance spectra (^13^C NMR) were recorded in CDCl_3_ on a Bruker Avance 400 or 500 MHz instrument, and tetramethylsilane (TMS) was used as the internal standard.

### General procedure for synthesis of compounds 2a, 2a′, 2b, 2b′, 2c and 2c′

To a solution of **1** (0.5 mmol) in dry DMF (5 mL) at 0–5 °C, a solution of *N*-chlorosuccinimide (NCS, 1.1 mmol) or *N*-bromosuccinimide (NBS, 1.1 mmol) or 1,3-dichloro-5,5-dimethylhydantoin (DCDMH, 1.1 mmol) in dry DMF (5 mL) was added dropwise. After adding, the mixture was stirred at 0–5 °C. When the reaction was complete, checked by TLC analysis, the reaction mixture was diluted with ice water (10 mL) and extracted with ethyl acetate (40 mL × 3). Subsequently, the combined organic phase was washed by saturated aqueous Na_2_CO_3_ (40 mL × 3) and brine (40 mL), dried over anhydrous Na_2_SO_4_, concentrated in vacuo, and purified by preparative thin-layer chromatography (PTLC) to give the pure products **2a, 2a’, 2b, 2b’, 2c** or **2c’**.

*Data for* **2a** (two isomers, α/β = 1.56/1 (C5′-Cl)). White solid, yield = 31%, m.p. 106–110 °C; [α]^20^_D_ = 15 (*c* 2.7 mg/mL, acetone); IR cm^−1^: 2948, 2919, 1775, 1752, 1208, 1046; ^1^H NMR (400 MHz, CDCl_3_) *δ*: 7.50 (t, *J* = 1.6 Hz, 1 H, H-4′), 6.68 (s, 0.38 H, H-5′), 6.64 (t, *J* = 1.6 Hz, 0.6 H, H-5′), 4.76 (s, 0.39 H, H-8), 4.72 (t, *J* = 1.6 Hz, 0.61 H, H-8), 2.25–2.32 (m, 1 H, H-4), 2.07–2.22 (m, 5 H, H-4, 5, 10), 1.83–1.85 (m, 1 H, H-6), 1.70–1.73 (m, 1 H, H-5), 1.52–1.60 (m, 1 H, H-6), 0.95 (s, 1.94 H, H-11), 0.90 (s, 1.24 H, H-11).

*Data for* **2a’.** White solid, yield = 17%, m.p. 102–104 °C; [α]^20^_D_ = −15 (*c* 3.1 mg/mL, acetone); ^1^H NMR (500 MHz, CDCl_3_) *δ*: 6.68 (d, *J* = 1.0 Hz, 1 H, H-4′), 4.80 (d, *J* = 1.0 Hz, 1 H, H-8), 2.21–2.34 (m, 3 H, H-4, 5), 2.19 (s, 3 H, H-10), 1.75–1.90 (m, 2 H, H-5, 6), 1.48–1.54 (m, 1 H, H-6), 1.14 (s, 3 H, H-11); ^13^C NMR (125 MHz, CDCl_3_) *δ*: 168.07, 151.16, 141.56, 133.87, 126.05, 111.65, 110.06, 80.95, 43.46, 32.09, 31.99, 20.59, 18.70, 18.27. HRMS (ESI): Calcd for C_14_H_15_O_3_Cl_4_ ([M + H]^+^), 370.9770; found, 370.9769.

*Data for* **2b**. Pale yellow solid, yield = 28%, m.p. 136–138 °C; [α]^20^_D_ = −16 (*c* 4.0 mg/mL, acetone); IR cm^−1^: 2953, 2923, 2850, 1785, 1746, 1207, 1042; ^1^H NMR (400 MHz, CDCl_3_) *δ*: 7.62 (t, *J* = 1.6 Hz, 1 H, H-4′), 6.93 (t, *J* = 1.6 Hz, 1 H, H-5′), 4.74 (t, *J* = 1.6 Hz, 1 H, H-8), 2.17–2.27 (m, 2 H, H-4), 2.13 (s, 3 H, H-10), 2.07–2.12 (m, 1 H, H-5), 1.83–1.85 (m, 1 H, H-6), 1.69–1.75 (m, 1 H, H-5), 1.52–1.59 (m, 1 H, H-6), 0.96 (s, 3 H, H-11). ^13^C NMR (125 MHz, CDCl_3_) *δ*: 168.52, 167.92, 151.02, 149.91, 130.77, 126.12, 81.32, 74.60, 43.35, 32.14, 31.98, 20.72, 18.65, 18.20. HRMS (ESI): Calcd for C_14_H_16_O_4_Br ([M + H]^+^), 327.0226; found, 327.0226.

*Data for* **2b’.** Pale yellow solid, yield = 18%, m.p. 98–100 °C; [α]^20^_D_ = −3 (*c* 4.3 mg/mL, acetone); ^1^H NMR (500 MHz, CDCl_3_) *δ*: 6.47 (s, 1 H, H-4′), 4.79 (d, *J* = 1.0 Hz, 1 H, H-8), 2.25–2.30 (m, 1 H, H-4), 2.13–2.19 (m, 4 H, H-4, 10), 1.66–1.85 (m, 3 H, H-5, 6), 1.46–1.52 (m, 1 H, H-6), 0.94 (s, 3 H, H-11); ^13^C NMR (125 MHz, CDCl_3_) *δ*: 169.32, 149.45, 126.55, 123.04, 122.69, 120.39, 114.05, 81.95, 44.29, 32.09, 32.00, 20.72, 18.51, 18.18. HRMS (ESI): Calcd for C_14_H_15_O_3_Br_2_ ([M + H]^+^), 388.9382; found, 388.9382.

*Data for* **2c** (two isomers, α/β = 1.8/1 (C5′-Cl)). White solid, yield = 21%, m.p. 106–110 °C; [α]^20^_D_ = 18 (*c* 2.9 mg/mL, acetone); ^1^H NMR (500 MHz, CDCl_3_) *δ*: 7.50 (t, *J* = 1.5 Hz, 1 H, H-4′), 6.68 (s, 0.35 H, H-5′), 6.64 ((t, *J* = 2.0 Hz, 0.65 H, H-5′), 4.76 (s, 0.36 H, H-8), 4.73 (t, *J* = 2.0 Hz, 0.66 H, H-8), 2.22–2.32 (m, 2 H, H-4), 2.14 (s, 3 H, H-10), 2.09–2.12 (m, 1 H, H-5), 1.84–1.88 (m, 1 H, H-6), 1.69–1.74 (m, 1 H, H-5), 1.54–1.59 (m, 1 H, H-6), 0.95 (s, 2 H, H-11), 0.90 (s, 1 H, H-11). ^13^C NMR (125 MHz, CDCl_3_) *δ*: 168.48, 168.38, 168.09, 167.99, 151.10, 151.06, 148.66, 148.43, 132.29, 132.07, 126.06, 125.98, 85.68, 85.40, 81.73, 43.04, 42.73, 32.13, 32.11, 31.97, 20.88, 20.73, 18.65, 18.23, 18.21.

*Data for* **2c’.** White solid, yield = 17%, m.p. 102–104 °C; [α]^20^_D_ = −19 (*c* 1.5 mg/mL, acetone); ^1^H NMR (500 MHz, CDCl_3_) *δ*: 6.33 (s, 1 H, H-4′), 4.82 (s, 1 H, H-8), 2.15–2.30 (m, 2 H, H-4), 2.13 (s, 3 H, H-10), 1.69–1.85 (m, 3 H, H-5, 6), 1.44–1.50 (m, 1 H, H-6), 0.94 (s, 3 H, H-11); ^13^C NMR (125 MHz, CDCl_3_) *δ*: 169.30, 149.49, 135.47, 132.04, 126.52, 118.24, 108.76, 81.45, 44.16, 32.09, 31.80, 20.68, 18.52, 18.17. HRMS (ESI): Calcd for C_14_H_15_O_3_Cl_2_ ([M + H]^+^), 301.0393; found, 301.0392.

### General procedure for synthesis of compound 3a–c and 3a’,b’

To a stirred suspension solution of AlCl_3_ (0.55 mmol) in dry CH_2_Cl_2_ (5 mL) at 25 °C, the corresponding acyl chloride (0.55 mmol) was added. The mixture was then stirred for 15 min, and a solution of **l** (0.5 mmol) in dry CH_2_Cl_2_ (5 mL) was added dropwise to the above mixture. When the reaction was complete, checked by TLC analysis, the reaction mixture was poured into ice water (15 mL) and extracted with CH_2_Cl_2_ (40 mL × 3). The combined organic phase was washed by saturated brine (40 mL), dried over anhydrous Na_2_SO_4_, concentrated in vacuo, and purified by PTLC to give the pure products **3a–c**, **3a’** or **3b’**.

*Data for* **3a**. White solid, yield = 57%, m.p. 182–184 °C; [α]^20^_D_ = −8 (*c* 3.8 mg/mL, acetone); IR cm^−1^: 2956, 2923, 2870, 1737, 1671, 1496, 1235, 908; ^1^H NMR (400 MHz, CDCl_3_) *δ*: 7.61 (s, 1 H, H-2′), 7.10 (s, 1 H, H-4′), 4.87 (s, 1 H, H-8), 2.48 (s, 3 H, -C*H*_3_), 2.21–2.32 (m, 2 H, H-4), 2.13 (s, 3 H, H-10), 1.71–1.84 (m, 3 H, H-5, 6), 1.43–1.51 (m, 1 H, H-6), 0.84 (s, 3 H, H-11); ^13^C NMR (100 MHz, CDCl_3_) *δ*: 187.00, 169.30, 153.42, 149.49, 143.26, 126.71, 123.49, 114.94, 82.48, 42.93, 32.05, 31.63, 26.04, 20.46, 18.52, 18.16. HRMS (ESI): Calcd for C_16_H_19_O_4_ ([M + H]^+^), 275.1278; found, 275.1278.

*Data for* **3a’.** White solid, yield = 32%, m.p. 116–118 °C; [α]^20^_D_ = 59 (*c* 5.3 mg/mL, acetone); IR cm^−1^: 2954, 2923, 2851, 1758, 1460, 1377, 997; ^1^H NMR (400 MHz, CDCl_3_) *δ: δ*: 7.48 (s, 1 H, H-5′), 6.69 (s, 1 H, H-4′), 5.67 (s, 1 H, H-8), 2.46 (s, 3 H, -C*H*_3_), 2.09–2.19 (m, 5 H, H-4, 10), 1.71–1.77 (m, 2 H, H-5, 6), 1.53–1.62 (m, 2 H, H-5, 6), 0.81 (s, 3 H, H-11); ^13^C NMR (100 MHz, CDCl_3_) *δ*: 188.70, 169.96, 149.35, 148.30, 144.73, 129.17, 127.04, 113.54, 81.39, 44.94, 32.18, 32.03, 26.92, 20.91, 18.52, 18.29. HRMS (ESI): Calcd for C_16_H_19_O_4_ ([M + H]^+^), 275.1278; found, 275.1277. *Data for* **3b.** White solid, yield = 42%, m.p. 106–108 °C; [α]^20^_D_ = −20 (*c* 3.5 mg/mL, acetone); IR cm^−1^: 2956, 2923, 2870, 1737, 1671, 1496, 1235, 908; ^1^H NMR (400 MHz, CDCl_3_) *δ*: 7.68 (s, 1 H, H-2′), 7.27 (s, 1 H, H-4′), 4.89 (s, 1 H, H-8), 4.59 (s, 2 H, -C*H*_2_Cl), 2.23–2.34 (m, 2 H, H-4), 2.14 (s, 3 H, H-10), 1.72–1.86 (m, 3 H, H-5, 6), 1.47–1.53 (m, 1 H, H-6), 0.84 (s, 3 H, H-11); ^13^C NMR (100 MHz, CDCl_3_) *δ*: 180.41, 169.16, 151.12, 149.79, 144.07, 126.51, 124.12, 116.47, 82.28, 45.14, 42.92, 32.05, 31.64, 20.51, 18.54, 18.14. HRMS (ESI): Calcd for C_16_H_18_O_4_Cl ([M + H]^ + ^), 309.0888; found, 309.0888.

*Data for* **3b’.** White solid, yield = 33%, m.p. 96–98 °C; [α]^20^_D_ = 45 (*c* 4.0 mg/mL, acetone); IR cm^−1^: 2946, 2918, 2872, 1757, 1687, 1472, 1203, 975; ^1^H NMR (400 MHz, CDCl_3_) *δ*: 7.55 (d, *J* = 1.6 Hz, 1 H, H-5′), 6.79 (d, *J* = 2.0 Hz, 1 H, H-4′), 5.66 (s, 1 H, H-8), 4.60–4.72 (m, 2 H, -C*H*_2_Cl), 2.19–2.26 (m, 2 H, H-4), 2.13 (s, 3 H, H-10), 1.77–1.83 (m, 2 H, H-5, 6), 1.58–1.63 (m, 2 H, H-5, 6), 0.84 (s, 3 H, H-11); ^13^C NMR (100 MHz, CDCl_3_) *δ*: 181.29, 169.70, 149.83, 146.06, 145.78, 132.03, 126.72, 114.04, 81.18, 46.13, 45.09, 32.20, 32.15, 20.98, 18.59, 18.28. HRMS (ESI): Calcd for C_16_H_18_O_4_Cl ([M + H]^+^), 309.0888; found, 309.0888.

*Data for* **3 c.** White solid, yield = 25%, m.p. 48–50 °C; [α]^20^_D_ = −17 (*c* 3.5 mg/mL, acetone); IR cm^−1^: 2953, 2926, 2857, 1758, 1675, 1457, 1204, 983; ^1^H NMR (400 MHz, CDCl_3_) *δ*: 7.60 (s, 1 H, H-2′), 7.09 (s, 1 H, H-4′), 4.88 (s, 1 H, H-8), 2.80 (t, *J* = 7.6 Hz, 2 H,-C*H*_2_(CH_2_)_4_CH_3_), 2.19–2.28 (m, 2 H, H-4), 2.14 (s, 3 H, H-10), 1.84–1.85 (m, 2 H, H-5, 6), 1.68–1.72 (m, 2 H, H-5, 6), 1.30–1.36 (m, 8 H, -CH_2_(C*H*_2_)_4_CH_3_), 0.86 (t, *J* = 6.8 Hz, 3 H,-CH_2_(CH_2_)_4_C*H*_3_), 0.84 (s, 3 H, H-11). HRMS (ESI): Calcd for C_21_H_29_O_4_ ([M + H]^+^), 345.2060; found, 345.2060.

### Biological assay

The insecticidal activity of **1**; **2a,a’,b,b’,c’**; and **3a,a’,b,b’,c** was tested as the mortality rate values by using the leaf-dipping method^13^, against the pre-third-instar larvae of *Mythimna separata*. For each compound, 30 pre-third-instar larvae (10 larvae per group) were used. Acetone solutions of **1**; **2a,a’,b,b’,c’**; **3a,a’,b,b’,c**; and toosendanin (a positive control) were prepared at 1 mg/mL. Fresh wheat leaf discs (1 × 1 cm) were dipped into the corresponding solution for 3 s, then taken out and dried. Leaf discs treated with acetone alone were used as a blank control group. Several pieces of treated leaf discs were kept in each dish (10 larvae were raised in each dish), which was then placed in a conditioned room (25 ± 2 °C, 65–80% relative humidity (RH), 12 h/12 h (light/dark) photoperiod). If the treated leaf discs were consumed, additional treated ones were added to the dish. After 48 h, untreated fresh leaves were added to all dishes until adult emergence. The corrected mortality rate values were obtained by the formula


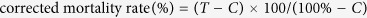


Where *T* is the mortality rate in the group treated with the tested compounds, and *C* is the mortality rate in the blank control group (*T* and *C* were all expressed as the percentage).

## Results and Discussion

**Figure 2:**
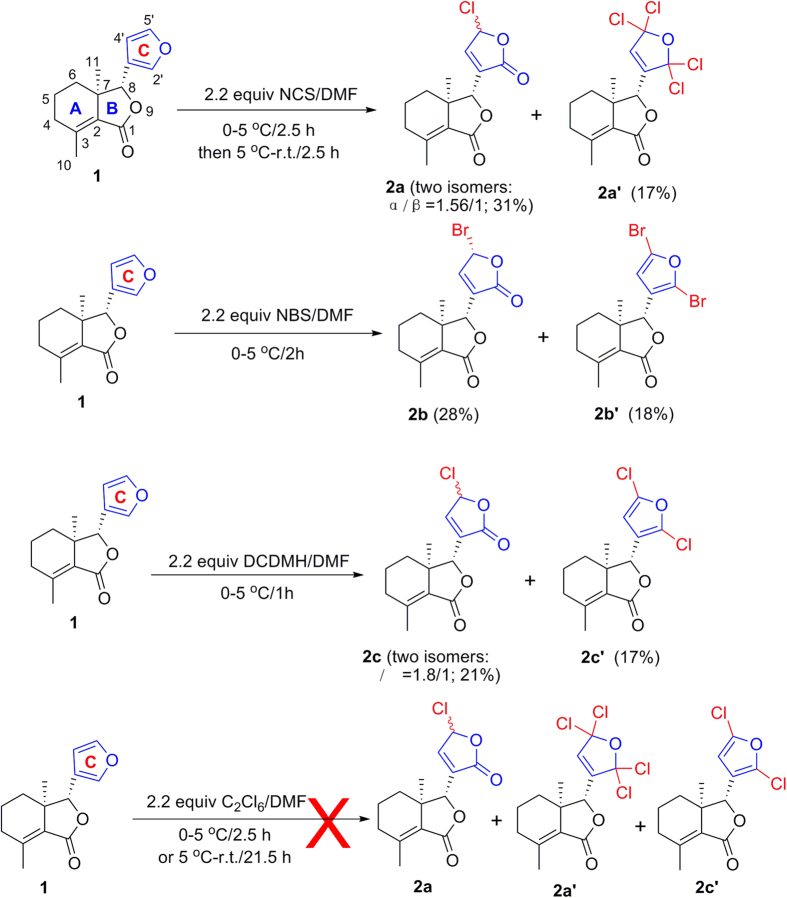
Investigation of halogenation of C-ring of fraxinellone.

**Figure 3:**
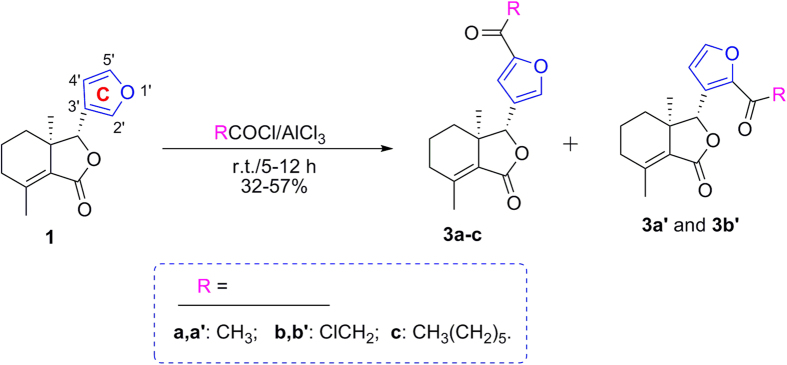
Investigation of acylation of C-ring of fraxinellone.

**Figure 4:**
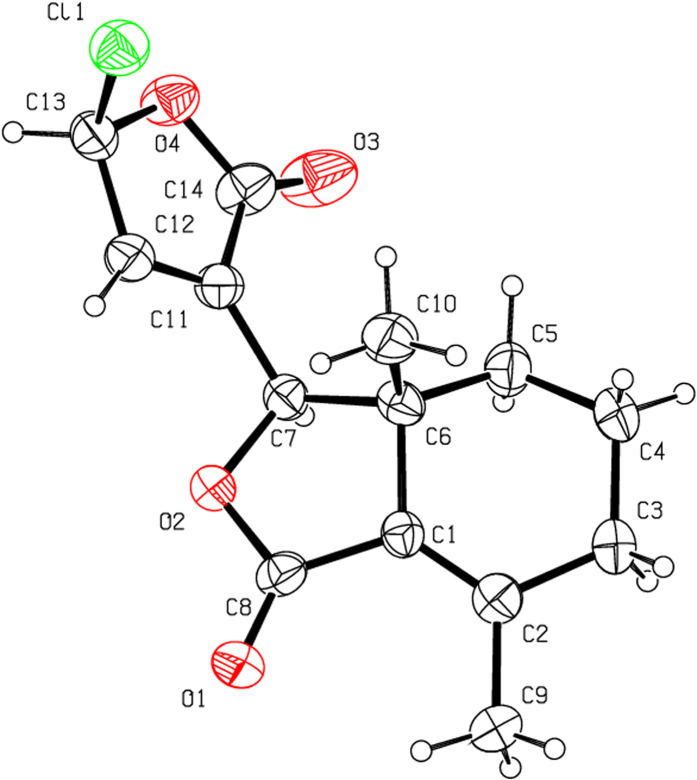
X-ray crystal structure of the 5′α-Cl isomer of 2a. Drawing by Hui Xu.

**Figure 5:**
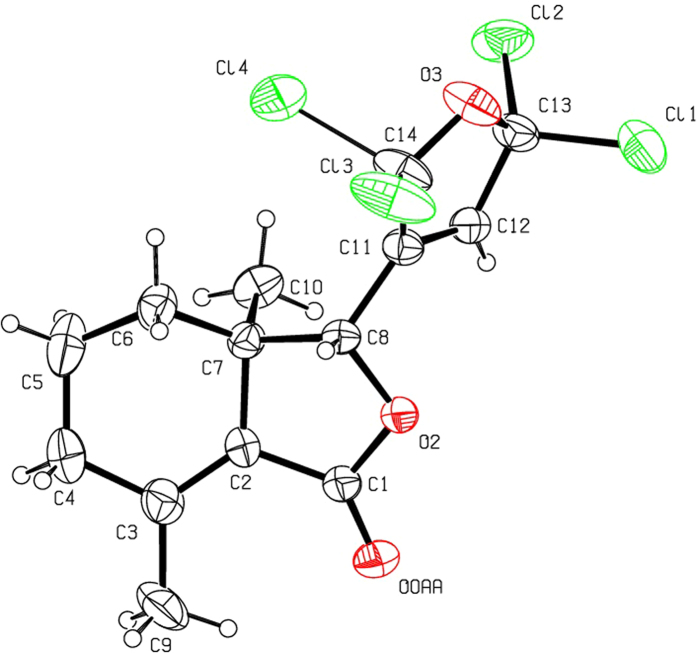
X-ray crystal structure of compound 2a’. Drawing by Hui Xu.

**Figure 6:**
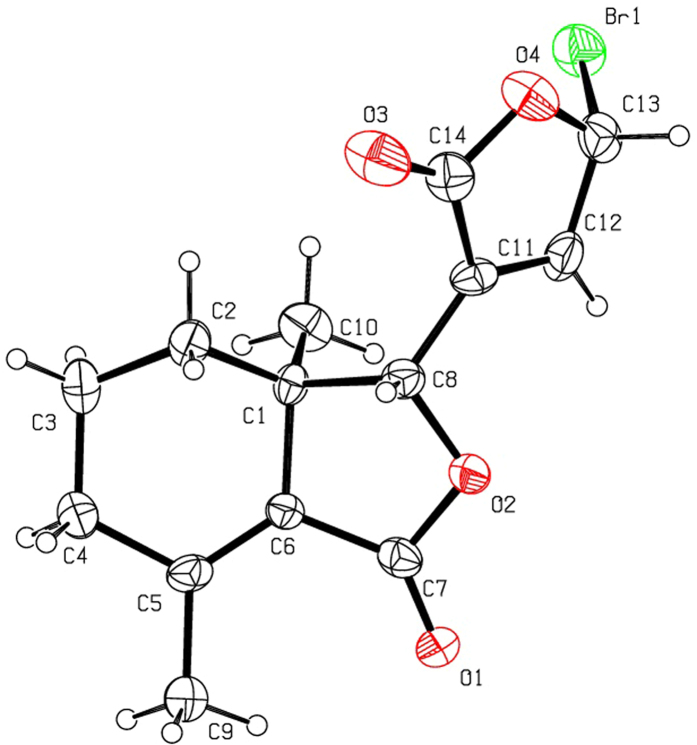
X-ray crystal structure of compound 2b. Drawing by Hui Xu.

**Figure 7:**
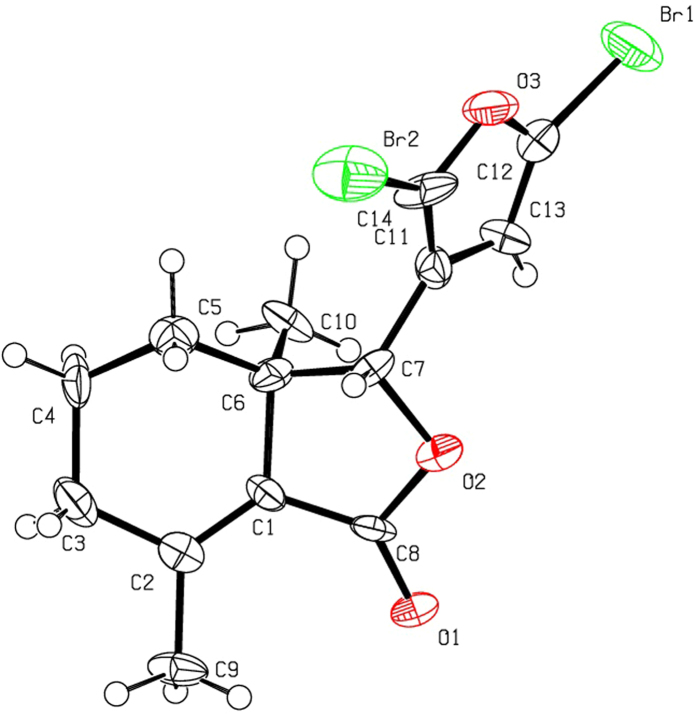
X-ray crystal structure of compound 2b’. Drawing by Hui Xu.

**Figure 8:**
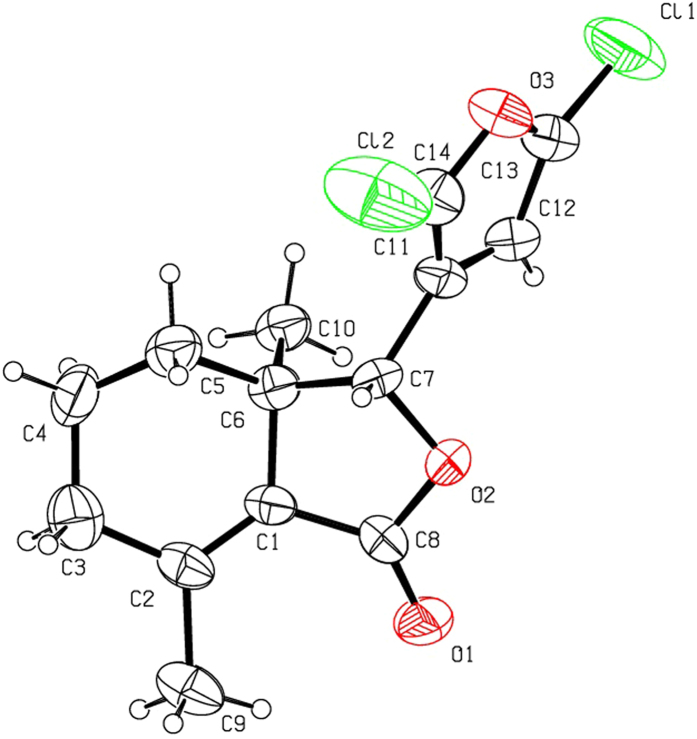
X-ray crystal structure of compound 2c’. Drawing by Hui Xu.

**Figure 9:**
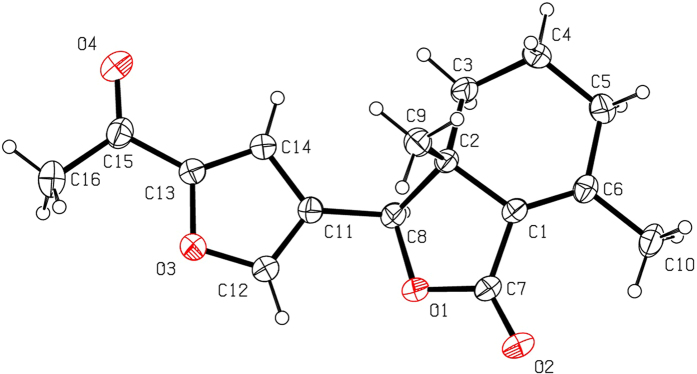
X-ray crystal structure of compound 3a. Drawing by Hui Xu.

**Figure 10:**
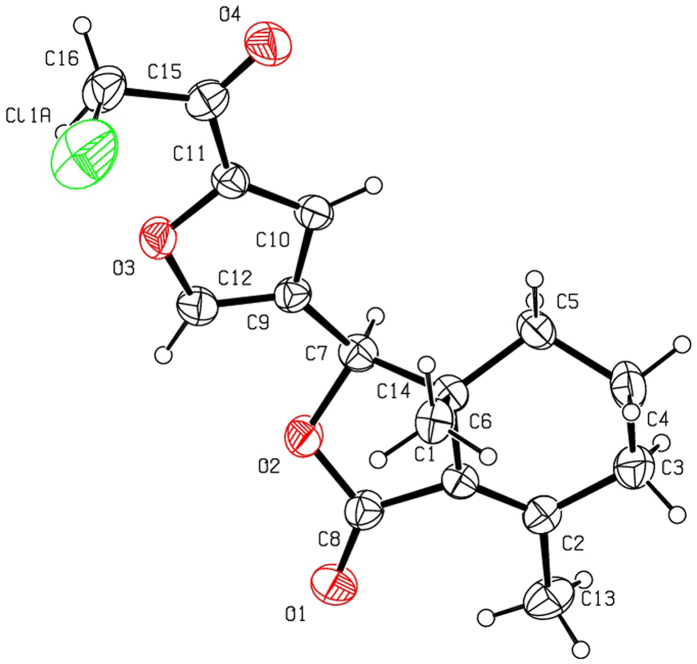
X-ray crystal structure of compound 3b. Drawing by Hui Xu.

**Figure 11:**
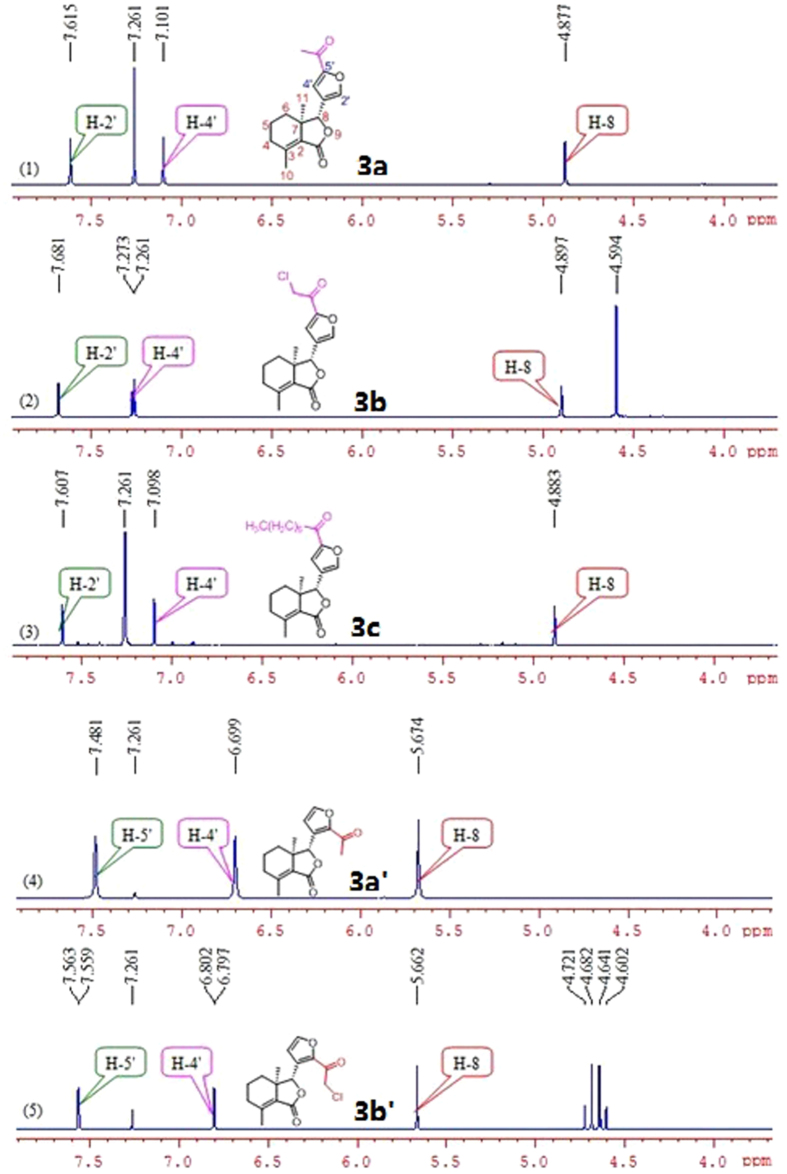
Comparsion of partial ^1^H NMR spectra of 3a–c and 3a’,b’.

Halogenation of C-ring of fraxinellone (**1**) with four different chlorination/bromination reagents was shown in Fig. 2. First, compound **1** reacted with 2.2 equiv. of *N*-chlorosuccinimide (NCS) to give 5′-chloro-substituted fraxinellone derivatives (**2a**, α/β = 1.56/1 (C5′-Cl)), and 2′,2′,5′,5′-tetrachlorofraxinellone (**2a’**). However, when compound **1** reacted with 2.2 equiv. of *N*-bromosuccinimide (NBS), 5′α-bromo-substituted fraxinellone derivative (**2b**), and 2′,5′-dibromofraxinellone (**2b’**) were produced. Subsequently, compound **1** reacted with 2.2 equiv. of 1,3-dichloro-5,5-dimethylhydantoin (DCDMH) to afford 5′-chloro-substituted fraxinellone derivatives (**2c**, α/β = 1.8/1 (C5′-Cl)), and 2′,5′-dichlorofraxinellone (**2c’**). Whereas compound **1** reacted with 2.2 equiv. of hexachloroethane (C_2_Cl_6_), no products were detected even if the reaction time was prolonged to 21.5 h at 5 °C to room temperature. In addition, acylation of C-ring of compound **1** was investigated as shown in Fig. 3. When compound **1** reacted with 1.1 equiv. of acetyl or chloroacetyl chloride in the presence of AlCl_3_, the corresponding 5′-acylfraxinellones (**3a** and **3b**) and 2′-acylfraxinellones (**3a’** and **3b’**) were produced. However, reaction of compound **1** with 1.1 equiv. of heptanoyl chloride only afforded 5′-heptanoylfraxinellone (**3c**). The structures of all target compounds were well characterized by ^1^H NMR (^13^C NMR), HRMS, optical rotation, IR, and mp. Copies of ^1^H NMR and ^13^C NMR spectra of 2a, a’, b, b’, c, c’; and 3a, a’, b, b’, c can be found in the Supporting Information. The steric configuration of **2a’**, **2b**, **2b’**, **2c’**, **3a**, **3b**, and one isomer (5′α-Cl) of **2a** was further determined by X-ray crystallography (Figs 4, 5, 6, 7, 8, 9 and 10). Crystallographic data (excluding structure factors) for the seven structures of **2a’**, **2b**, **2b’**, **2c’**, **3a**, **3b**, and one isomer (5’α-Cl) of **2a** have been deposited with the Cambridge Crystallographic Data Centre as supplementary publication numbers CCDC 1478207–1478213, respectively. These data can be obtained free of charge on application to CCDC [fax + 44 (0)1223 336033 or e-mail deposit@ccdc.cam.ac.uk]. As depicted in the partial ^1^H NMR spectra of Fig. 11, the chemical shifts of H-4′ and H-8 of **3a** and **3b** were about 7.1–7.2, and 4.9 ppm, respectively; whereas the chemical shifts of H-4′ and H-8 of **3a’** and **3b’** were about 6.7–6.8, and 5.7 ppm, respectively. For the chemical shifts of H-4′ and H-8 of **3c** were 7.1 and 4.9 ppm, respectively, the heptanoyl group of **3c** was at its C-5′ position.

**Figure 12:**
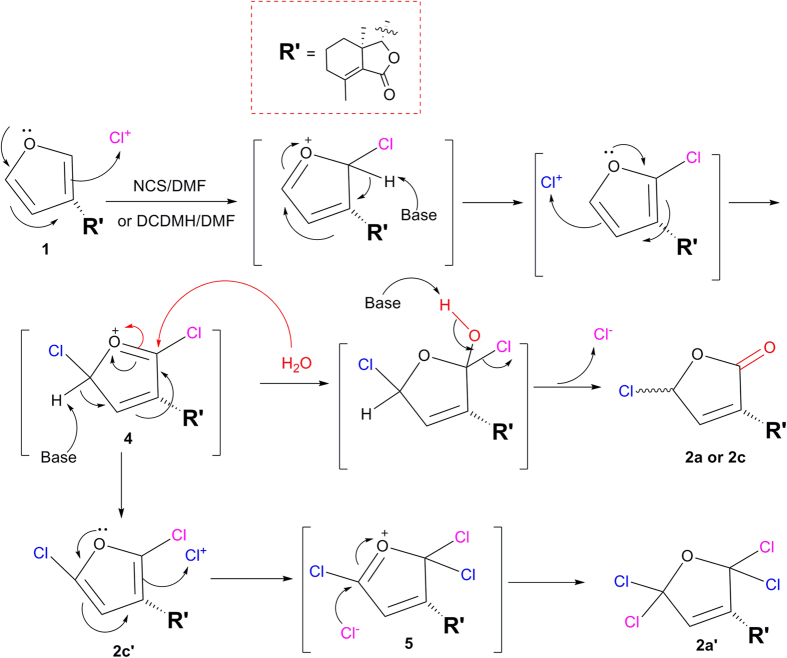
Possible mechanism for NCS or DCDMH reaction with 1.

The possible mechanism for NCS or DCDMH reaction with **1** was described in Fig. 12. If the key intermediate **4** reacted with water, compounds **2a** or **2c** were produced. Additionally, the key intermediate **4** reacted with a base to give **2c’**, which further reacted with a Cl^ + ^ion to afford the intermediate **5**. Finally, compound **2a’** was produced by reaction of **5** with a Cl^−^ anion. The mechanism for NBS reaction with **1** was similar to that of NCS. If the bromine atom was at the 5′β position on the furyl ring, there will be a big steric effect between the bromine atom and the hydrogen atom at the C-8 position. So the bromine atom at the 5′α position on the furyl ring of **2b** was reasonable.

As shown in Table 1, the insecticidal activity of **1**; **2a,a’,b,b’,c’**; and **3a,a’,b,b’,c** against the pre-third-instar larvae of *M. separata in vivo* was evaluated as the mortality rates at 1 mg/mL. Toosendanin was used as the positive control at 1 mg/mL, and leaves treated with acetone alone were used as a blank control group. As our previous reports^13^,^14^,^15^, the prepared halogenation/acylation derivatives of fraxinellone (**2a,a’,b,b’,c’**; and **3a,a’,b,b’,c**) also exhibited the delayed insecticidal activity against *M. separata*. For example, the corrected mortality rates of **3c** against *M. separata* after 10 and 20 days were 16.7% and 23.3%, respectively. However, it was sharply increased to 53.6% after 35 days, which was greater than 3-fold of that after 10 days. Among all derivatives, compounds **2a**, **2a’**, **3a** and **3c** displayed more potent insecticidal activity than their precursor **1** and toosendanin (a positive control). For example, the final mortality rates of **2a**, **2a’**, **3a** and **3c** were 50.9%, 51.9%, 57.1% and 53.6%, respectively; whereas the final mortality rates of **1** and toosendanin were 42.9% and 46.4%, respectively. Generally, introduction of the halogen atoms at the furyl ring of **1** could not result in more potent compounds (e.g., **2b’** and **2c’** *vs* **1**). However, when the furyl ring of **1** was converted to the lactone or dihydrofuryl ring, the corresponding compounds **2a**, **2b**, and **2a’** exhibited the insecticidal activity equal to, or higher than, that of **1**. In general, introducing the acyl group at the furyl ring of **1** was favorable for the insecticidal activity when compared with introduction of the halogen atom at the furyl ring of **1** (e.g., **2b’** and **2c’** *vs* **3a–c**). Introduction of the acyl group at the C-5′ position of C-ring of **1** gave the more promising compound than that containing the same group at its C-2′ position (**3a** *vs* **3a’**; **3b** *vs* **3b’**). For example, the final mortality rates of **3a** and **3b** were 57.1% and 46.4%, respectively; whereas the final mortality rates of **3a’** and **3b’** were 46.4% and 35.7%, respectively.

## Conclusion

In summary, halogenation/acylation of the furyl-ring of fraxinellone was investigated. We have developed halogenation of fraxinellone with different chlorination/bromination reagents. Their possible reaction mechanism was also proposed. For the limonoids containing the furyl-ring, the present method could be applied to halogenate their furyl-ring to give many halogenation products. Seven key steric structures of **2a’**, **2b**, **2b’**, **2c’**, **3a**, **3b**, and one isomer (5’α-Cl) of **2a** were confirmed by single-crystal X-ray diffraction. Especially compounds **2a**, **2a’**, **3a** and **3c** displayed more potent insecticidal activity than their precursor and toosendanin. It suggested that introducing the acyl group at the furyl ring of fraxinellone was more favorable for the insecticidal activity when compared with introduction of the halogen atom at the same position. It will pave the way for further design and chemical modifications of fraxinellone as botanical insecticidal agents.

## Additional Information

**How to cite this article**: Guo, Y. *et al*. New Insecticidal Agents from Halogenation/Acylation of the Furyl-Ring of Fraxinellone. *Sci. Rep.* **6**, 35321; doi: 10.1038/srep35321 (2016).

## Supplementary Material

Supplementary Information

## Figures and Tables

**Figure 1:**
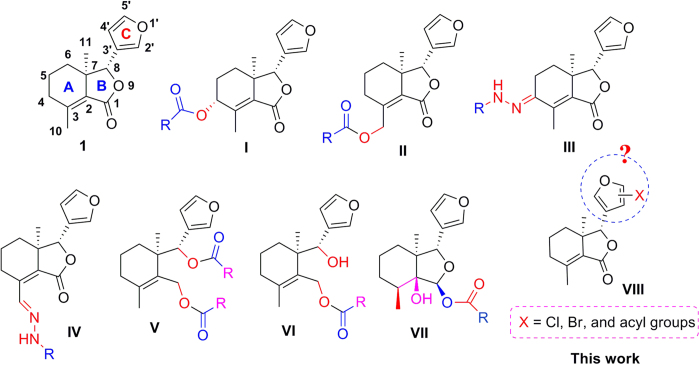
Chemical structures of fraxinellone (1) and its derivatives (I-VIII).

**Table 1:** Insecticidal Activity of Halogenation and Acylation Products of Fraxinellone against *M. separata* on Leaves Treated with a Concentration of 1 mg/mL.

**Compound**	**Corrected mortality rate (%)**		
	**10 days**	**20 days**	**35 days**
**1**	10.0 ± 0	13.3 ± 3.3	42.9 ± 3.3
**2a**	13.3 ± 3.3	26.7 ± 3.3	50.9 ± 3.3
**2a′**	10.0 ± 0	23.3 ± 3.3	51.9 ± 3.3
**2b**	16.7 ± 3.3	23.3 ± 3.3	42.9 ± 3.3
**2b′**	6.7 ± 3.3	16.7 ± 3.3	37.7 ± 3.3
**2c′**	6.7 ± 3.3	20.0 ± 0	40.7 ± 3.3
**3a**	20.0 ± 5.8	23.3 ± 3.3	57.1 ± 0
**3a′**	36.7 ± 3.3	40.0 ± 0	46.4 ± 5.8
**3b**	16.7 ± 3.3	23.3 ± 3.3	46.4 ± 5.8
**3b′**	13.3 ± 3.3	13.3 ± 3.3	35.7 ± 0
**3c**	16.7 ± 3.3	23.3 ± 3.3	53.6 ± 3.3
toosendanin	6.7 ± 3.3	16.7 ± 3.3	46.4 ± 5.8
blank control	0 ± 0	0 ± 0	6.7 ± 3.3
