# Supplementary material for: New Insecticidal Agents from Halogenation/Acylation of the Furyl-Ring of Fraxinellone
Source: Sci Rep. 2016 Oct 24;6:35321. doi: 10.1038/srep35321 (PMC5075773; doi:10.1038/srep35321)

New Insecticidal Agents from Halogenation/Acylation of the Furyl-Ring of Fraxinellone

Yong Guo^1,2,†^, Ruige Yang^1,†^ & Hui Xu^1^

^1^Research Institute of Pesticidal Design & Synthesis, College of Sciences/Plant Protection, Northwest A&F University, Yangling 712100, Shaanxi Province, P. R. China.

^2^Collaborative Innovation Center of New Drug Research and Safety Evaluation (Henan Province), School of Pharmaceutical Sciences, Zhengzhou University, Zhengzhou 450001, Henan Province, P. R. China.

Correspondence and requests for materials should be addressed to H.X. ([orgxuhui@nwsuaf.edu.cn](mailto:orgxuhui@nwsuaf.edu.cn)); Telephone: +86(0)29-87091952; Fax: +86(0)29-87091952.

^†^These authors contributed equally to this work.

Copies of ^1^H NMR and ^13^C NMR spectra ……..…….……………………..**2-20**

Data for **2a** (two isomers: α/β=1.56/1)


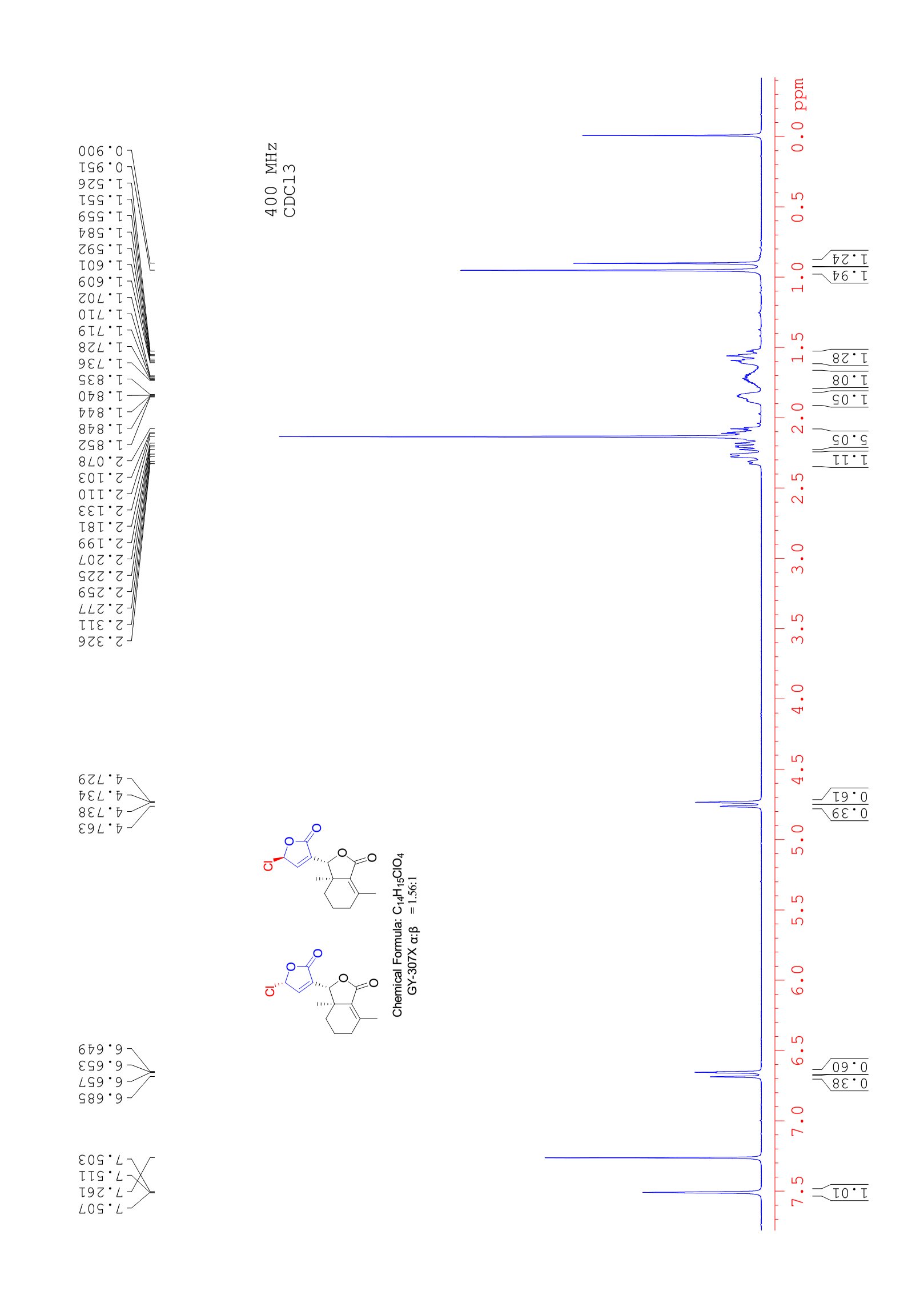


Data for **2a′**:


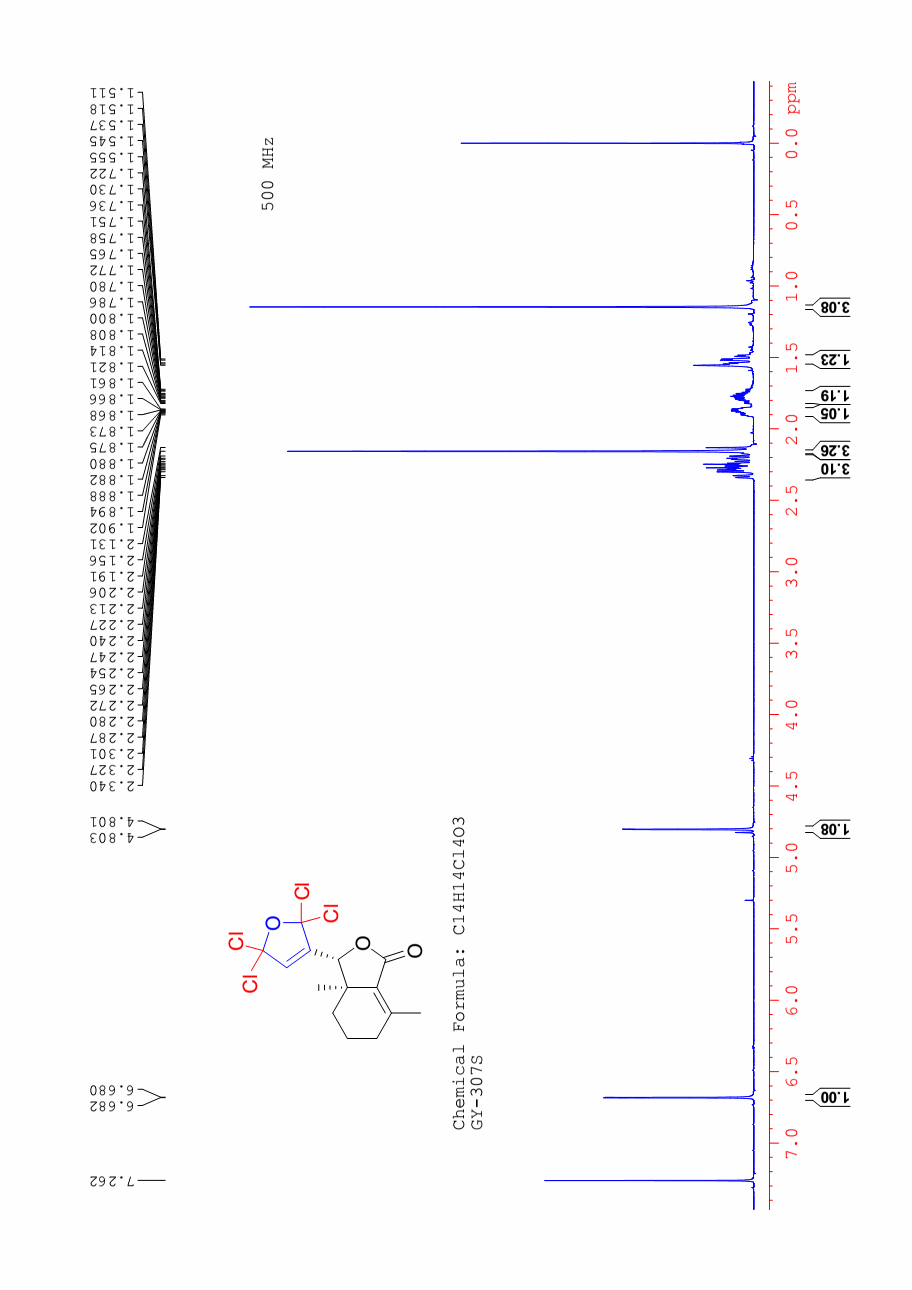


Data for **2a′**:


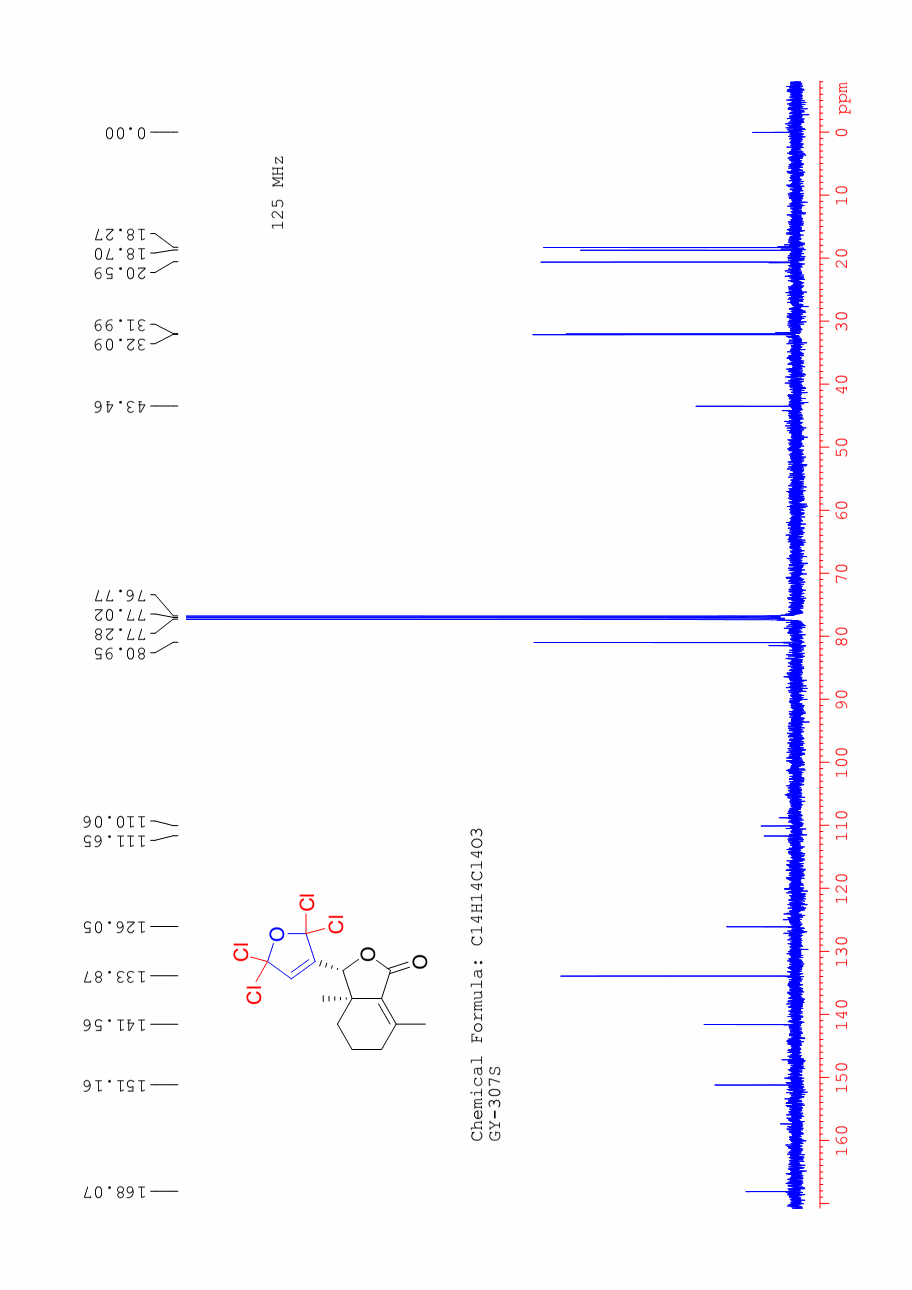


Data for **2b**:


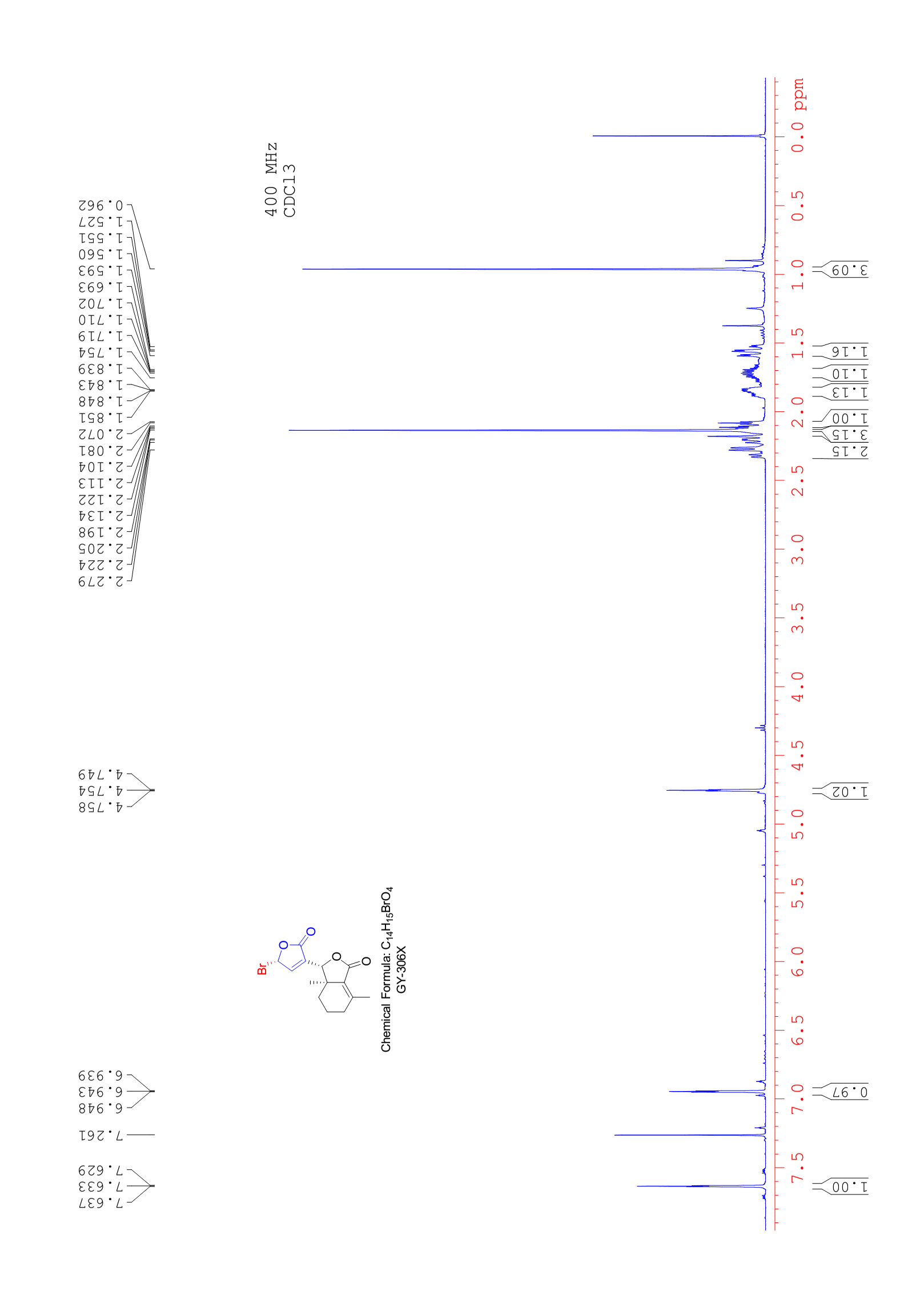


Data for **2b**:


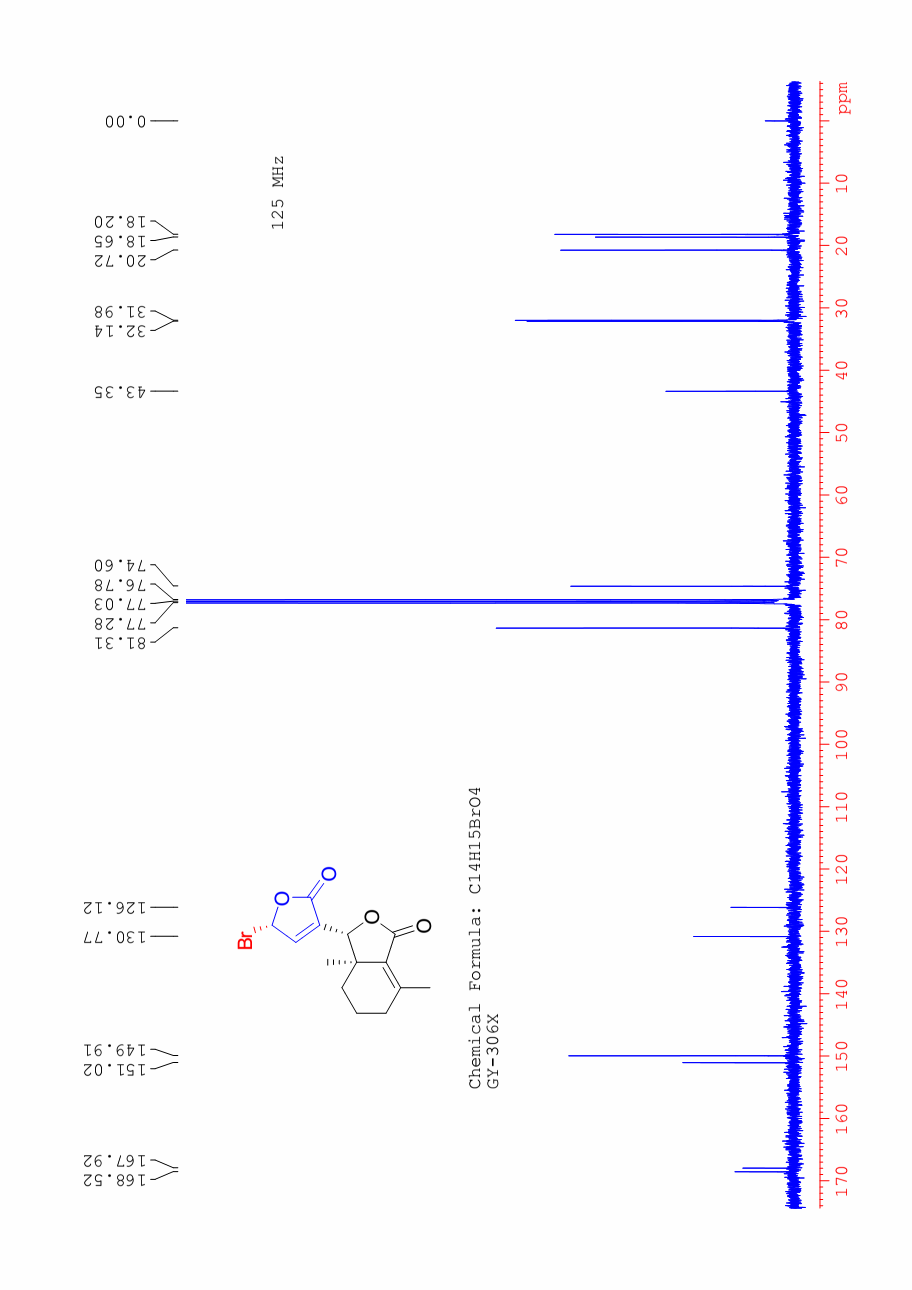


Data for **2b′**


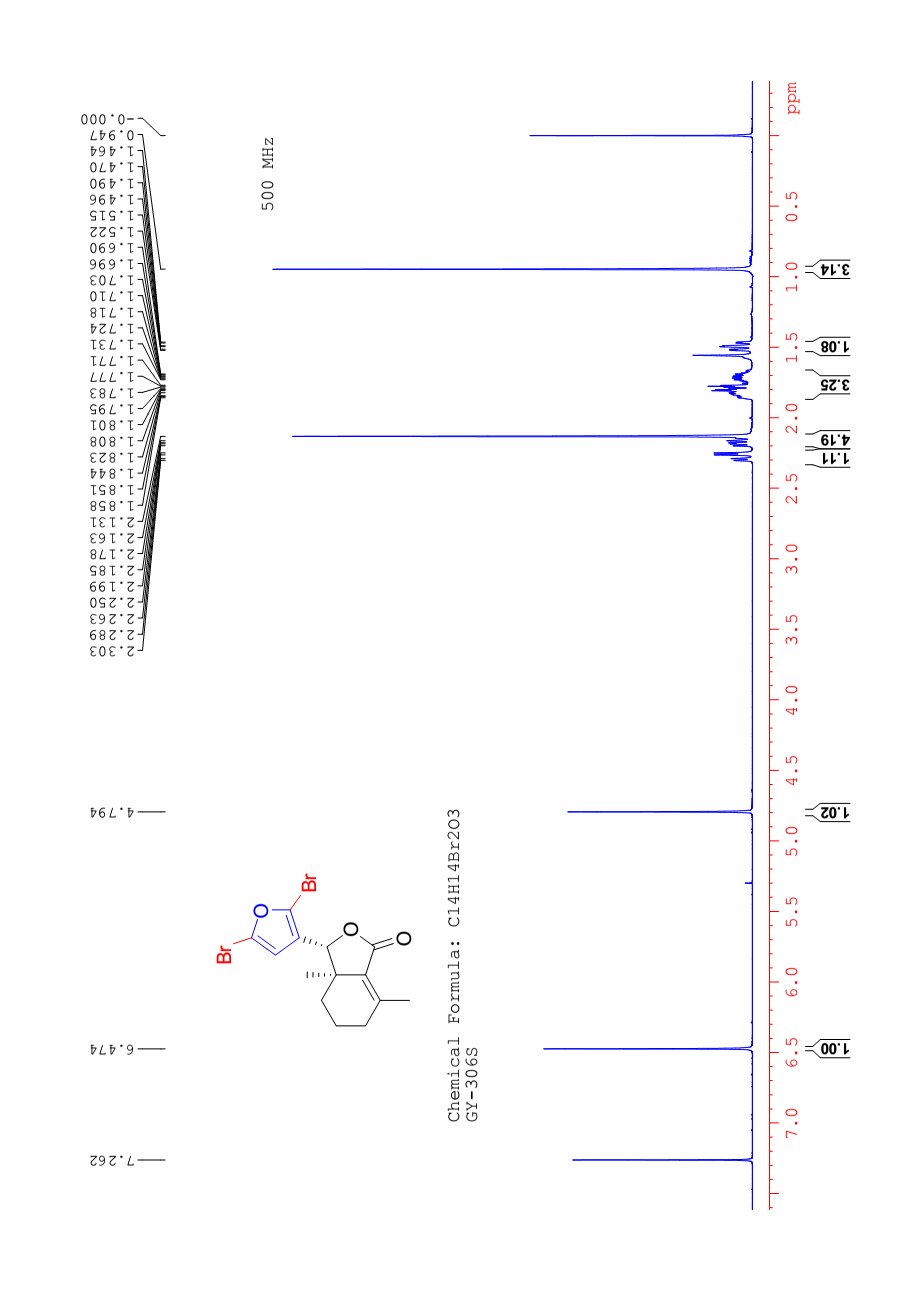


Data for **2b′**


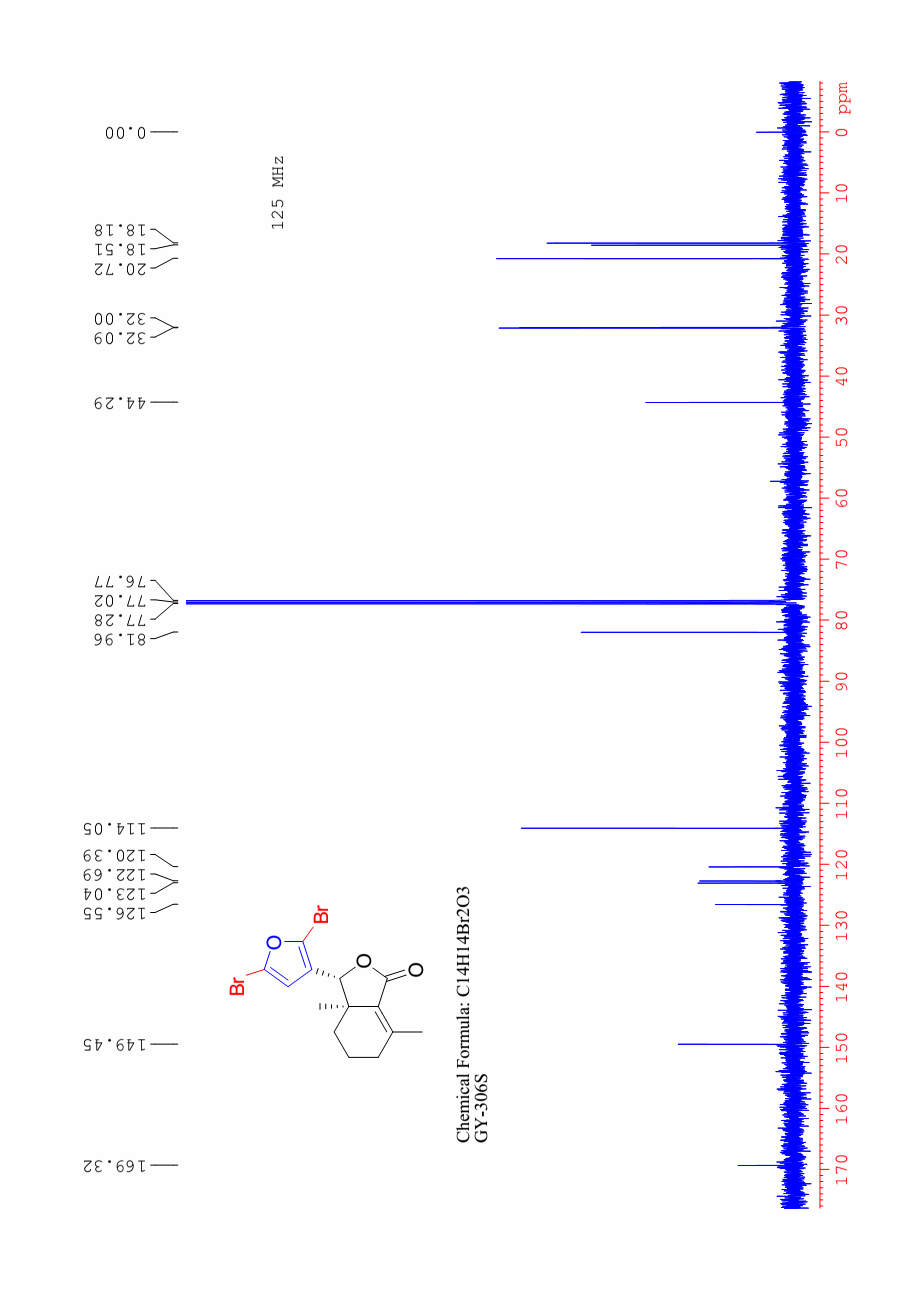


Data for **2c**(two isomers: α/β=1.8/1)


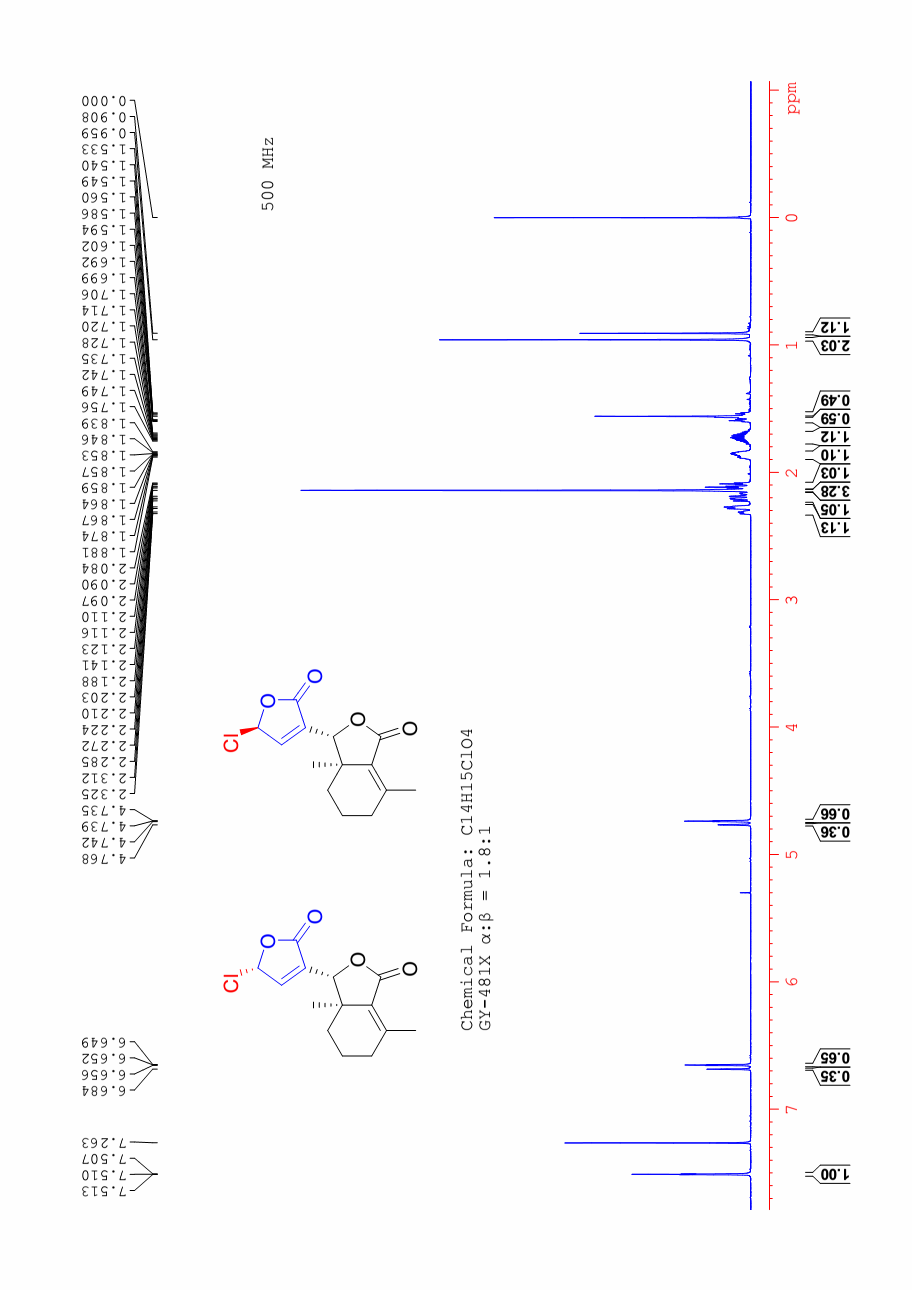


Data for **2c′**:


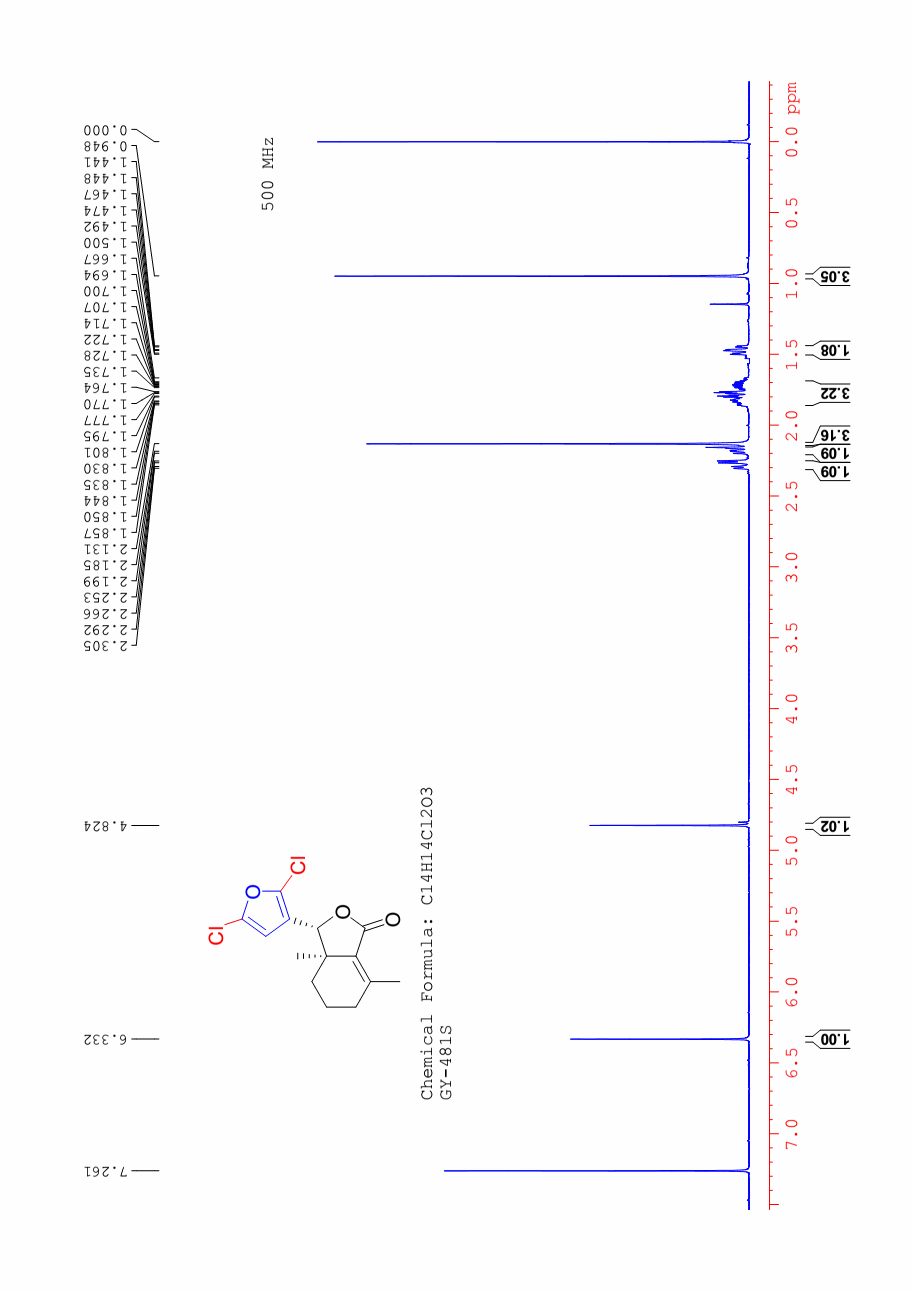


Data for **2c′**:


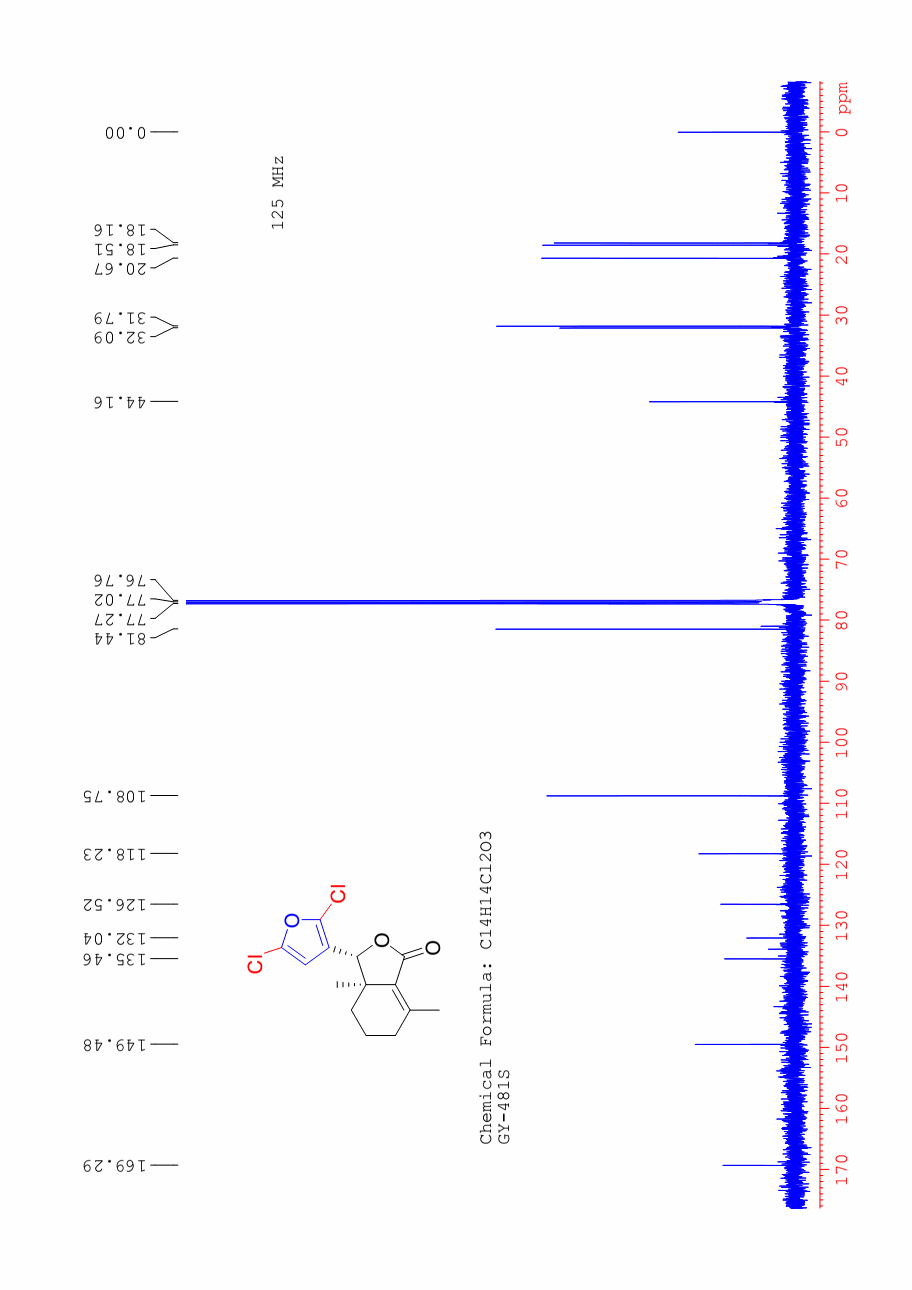


Data for **3a**:


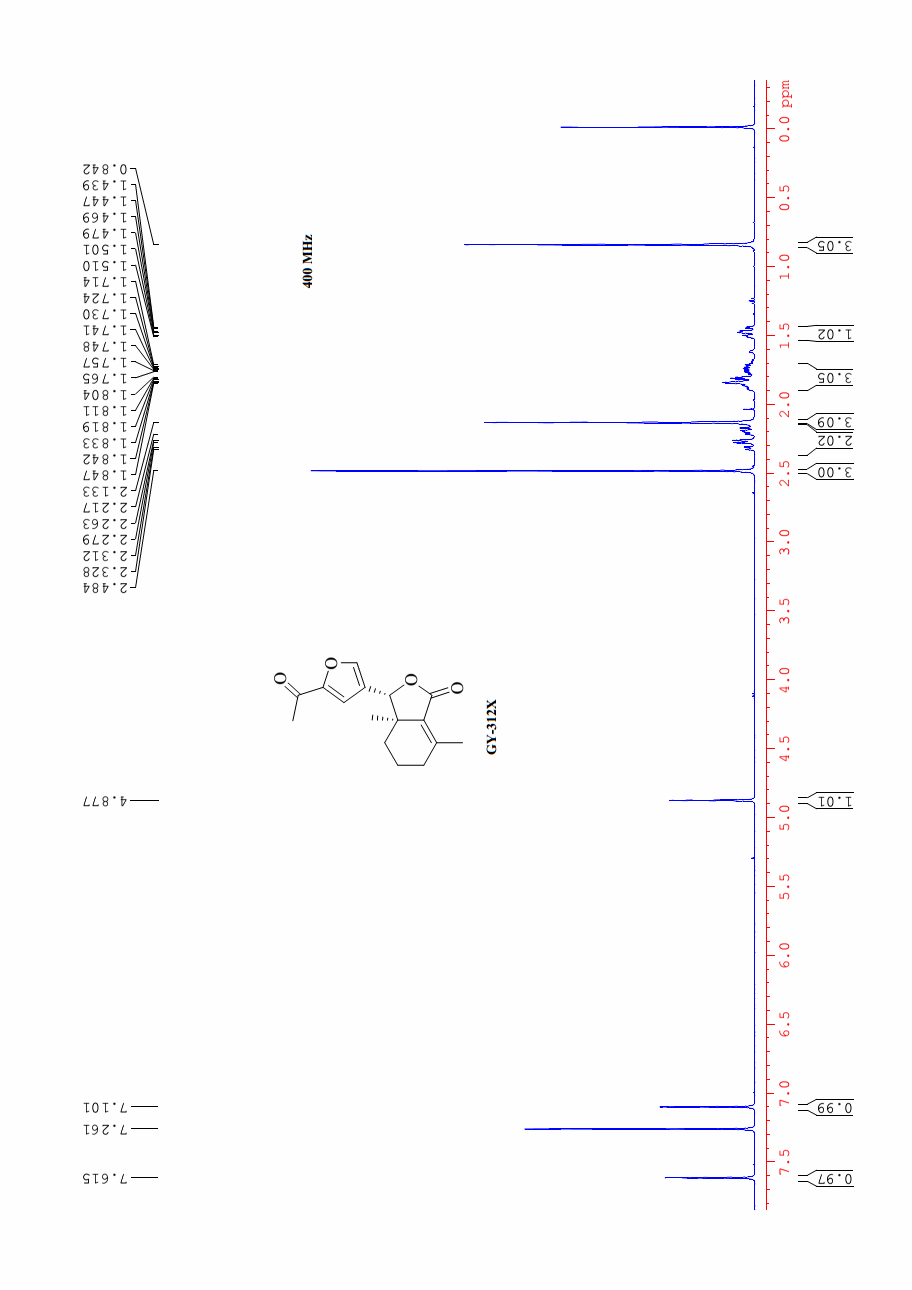


Data for **3a**:


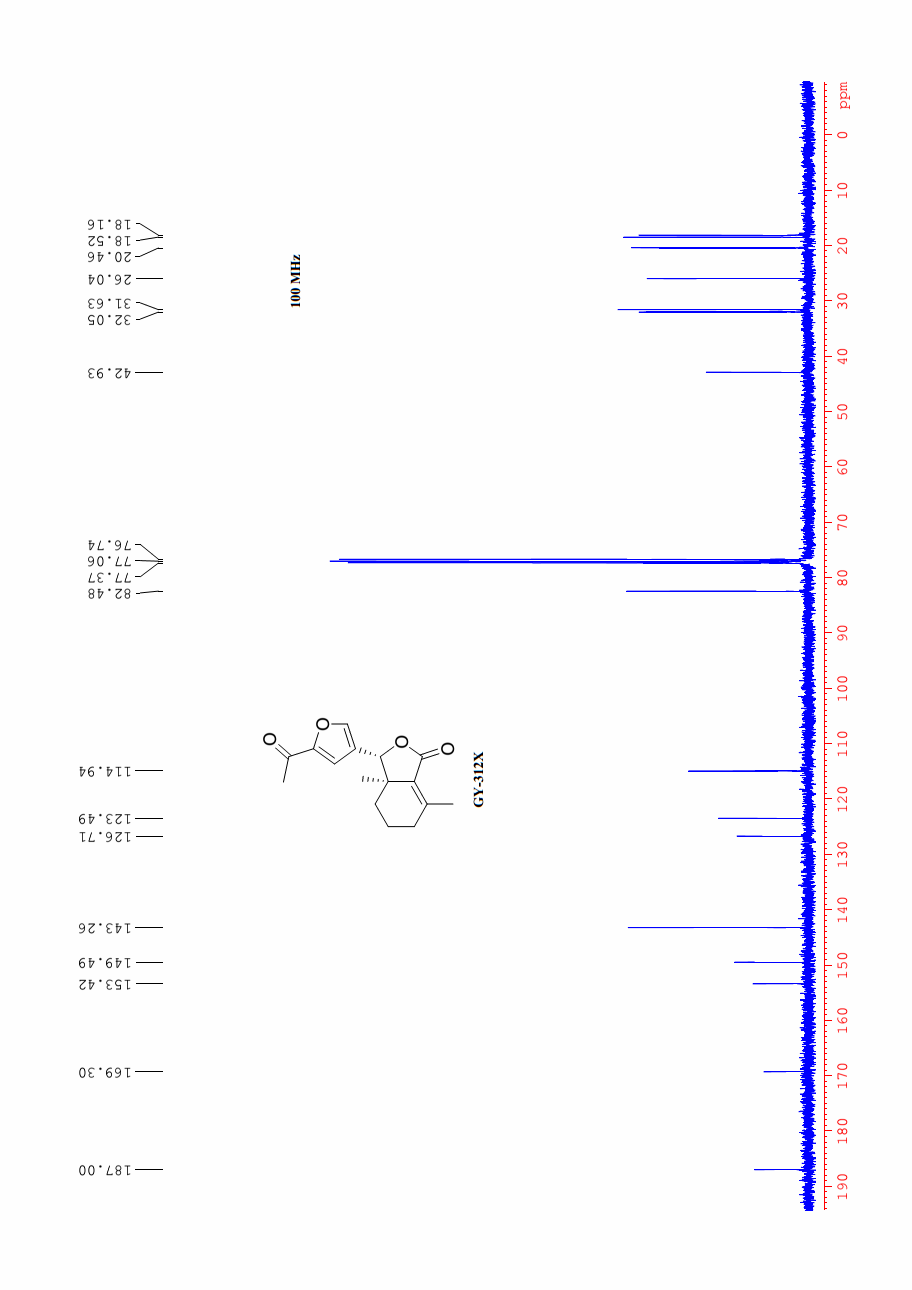


Data for **3a'**:


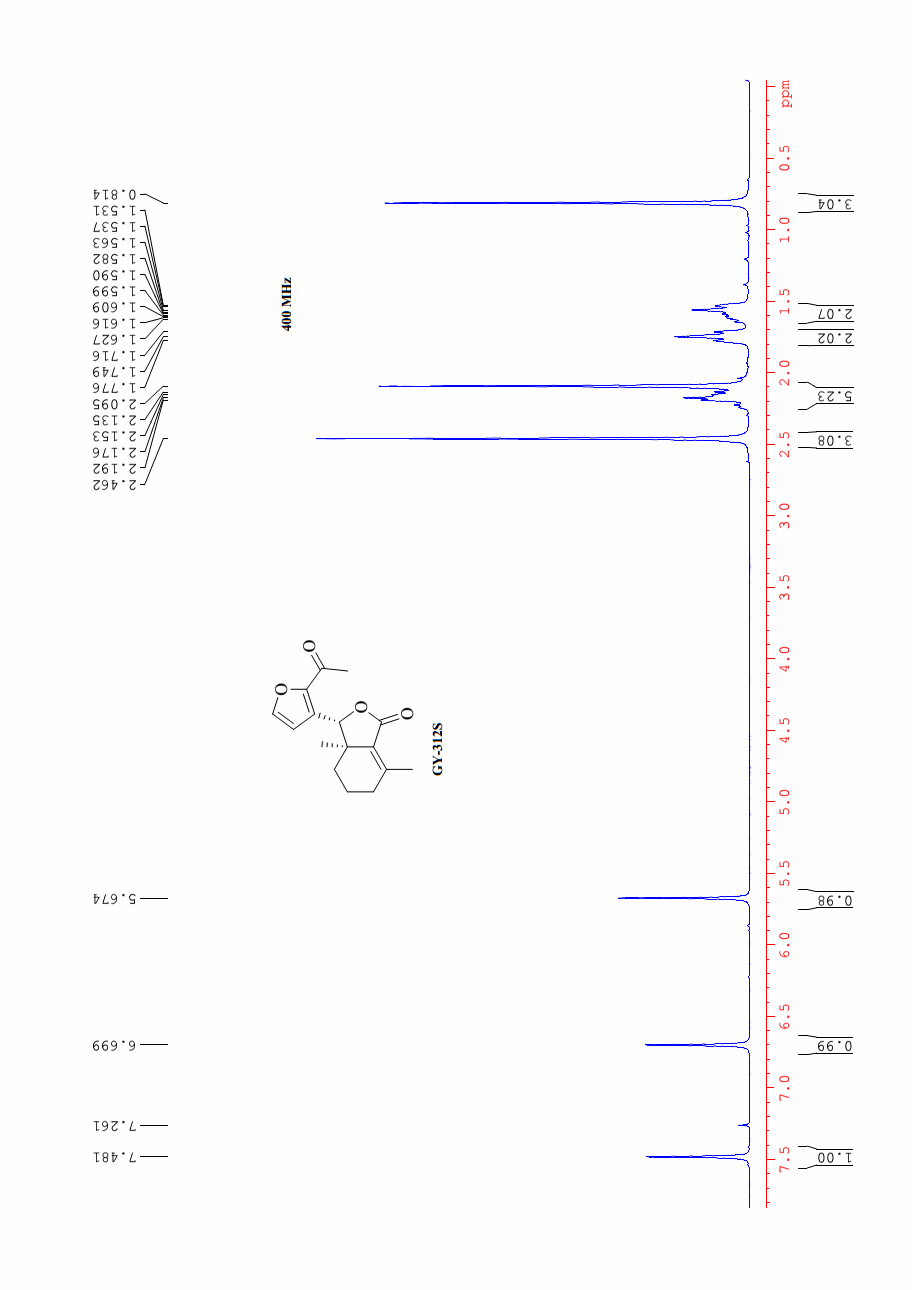


Data for **3a'**:


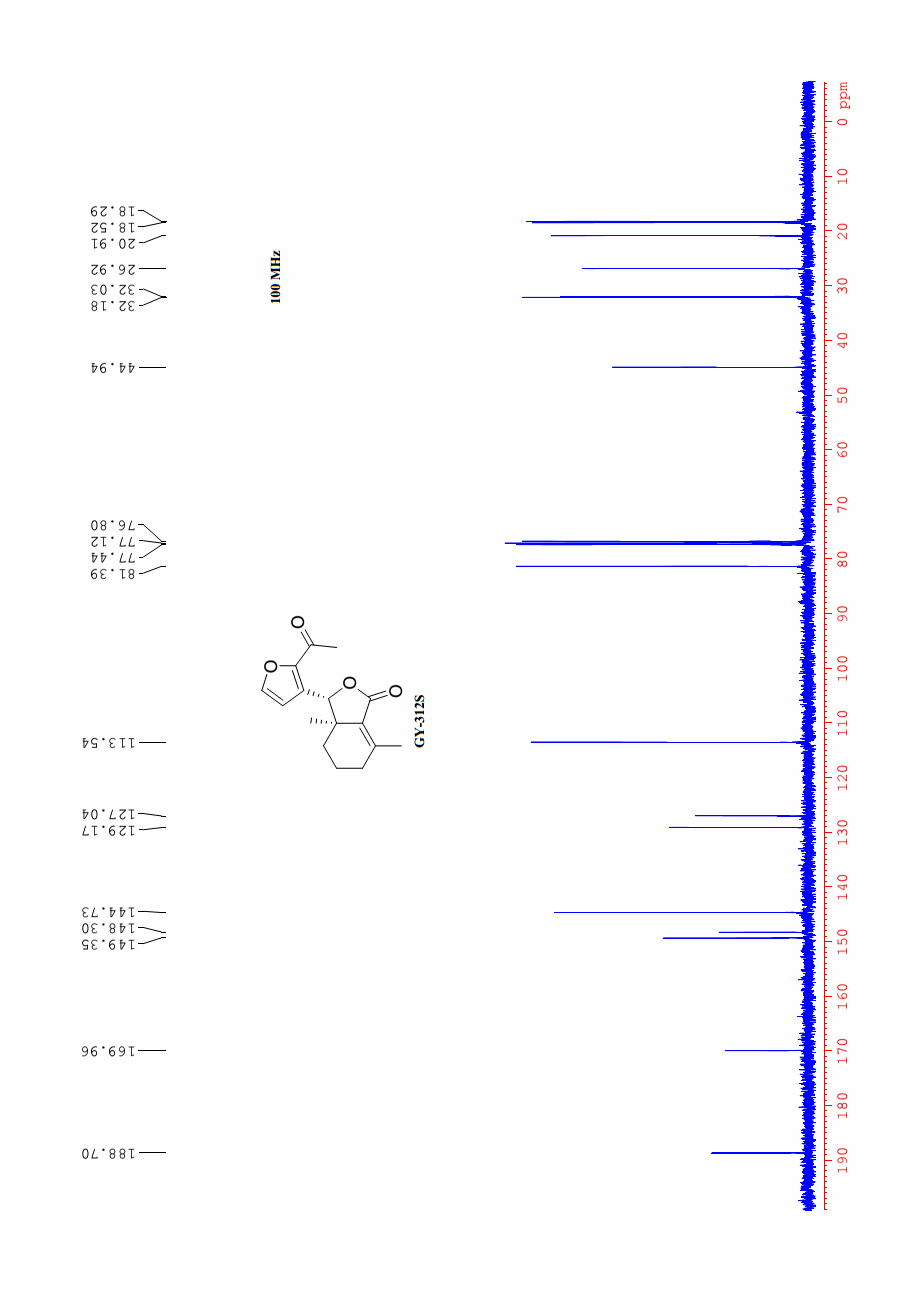


Data for **3b**:


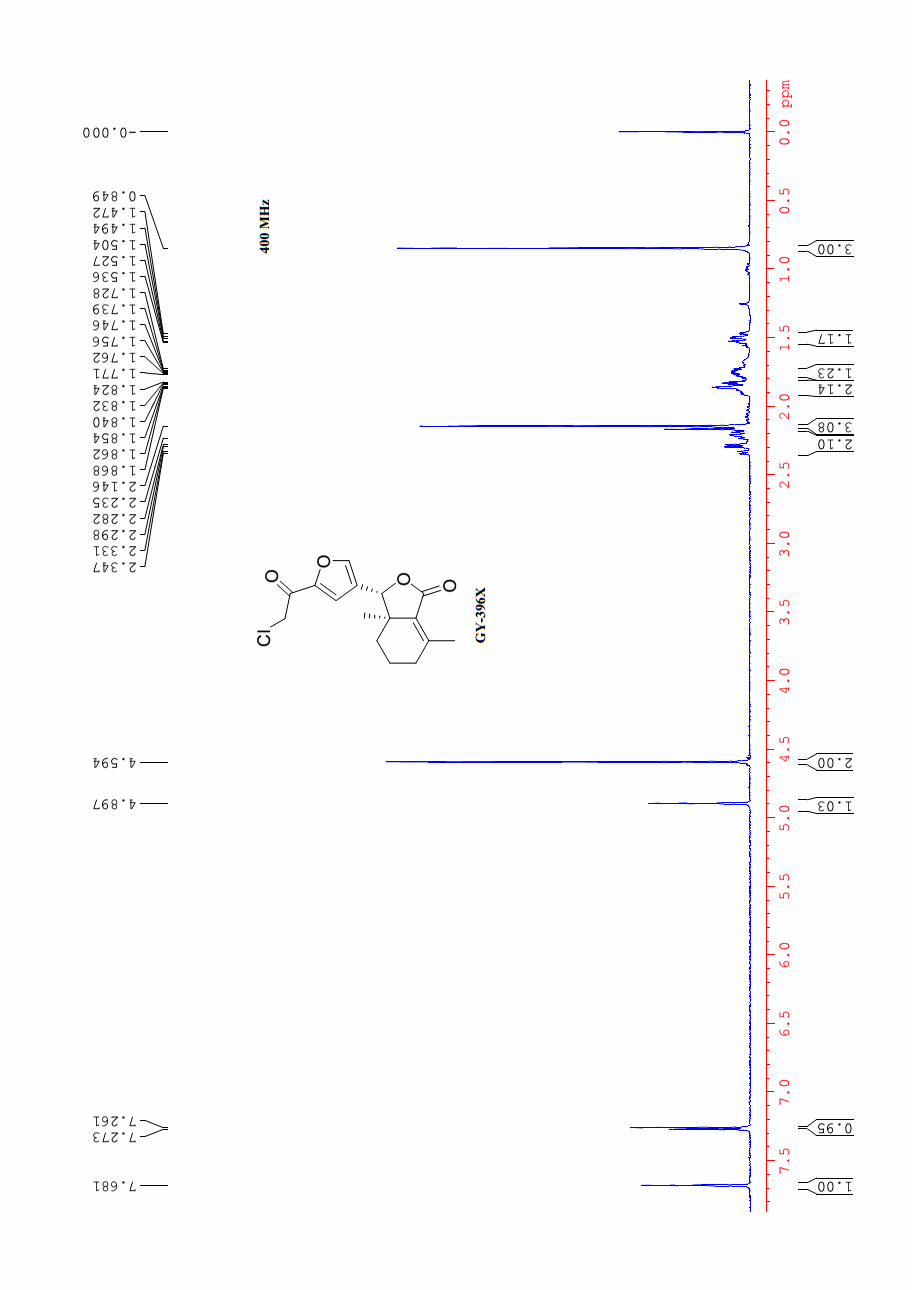


Data for **3b**:


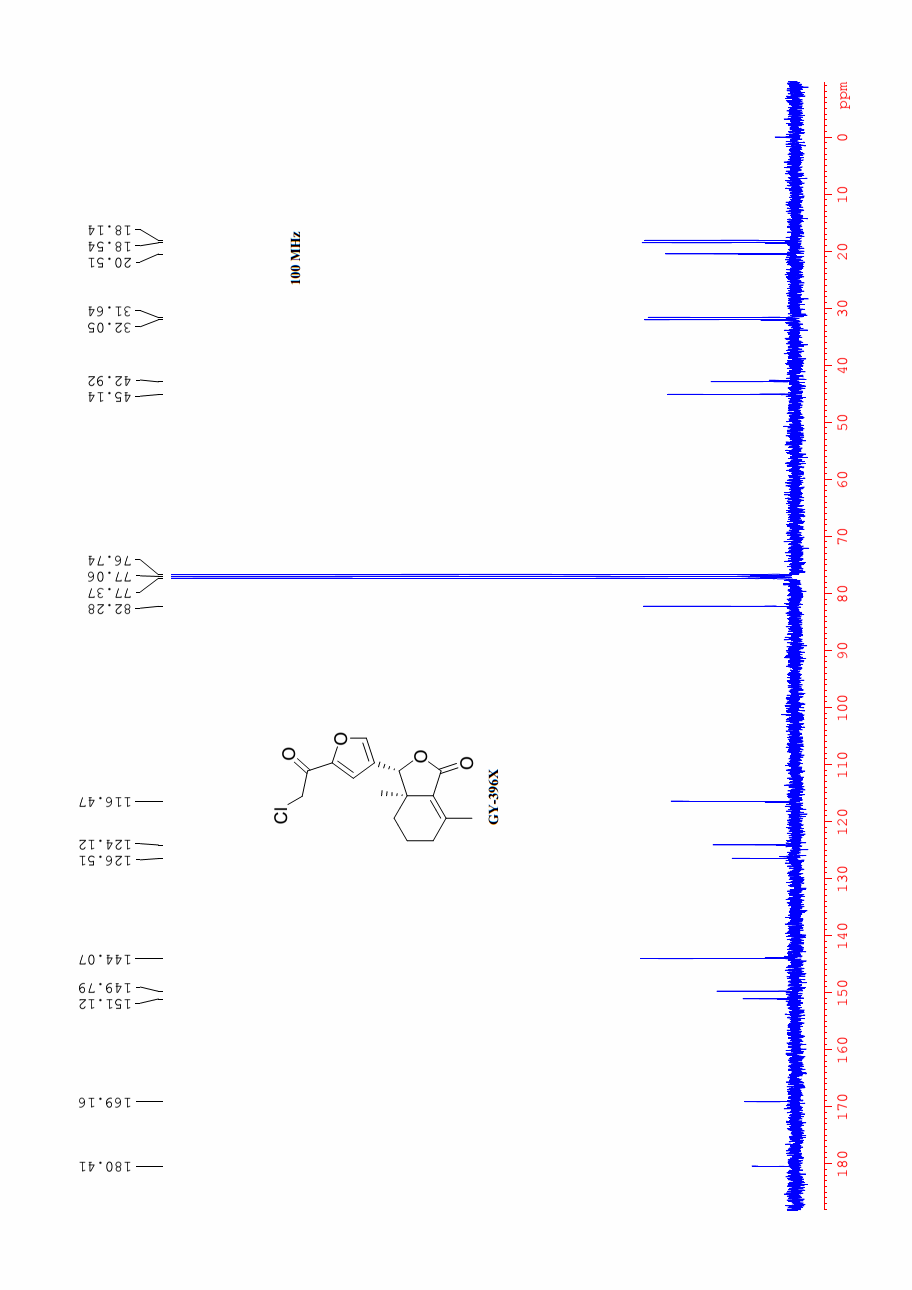


Data for **3b'**:


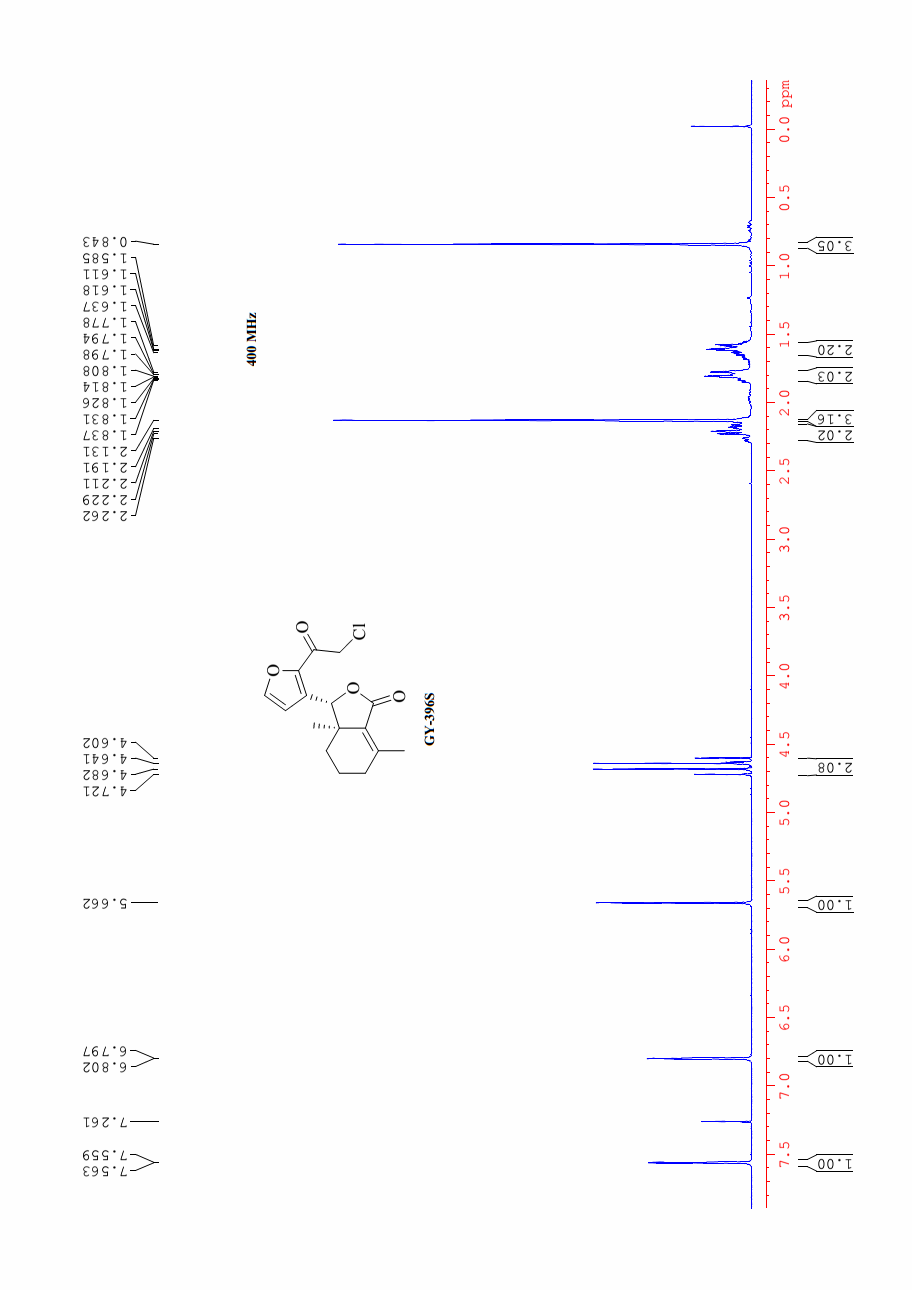


Data for **3b'**:


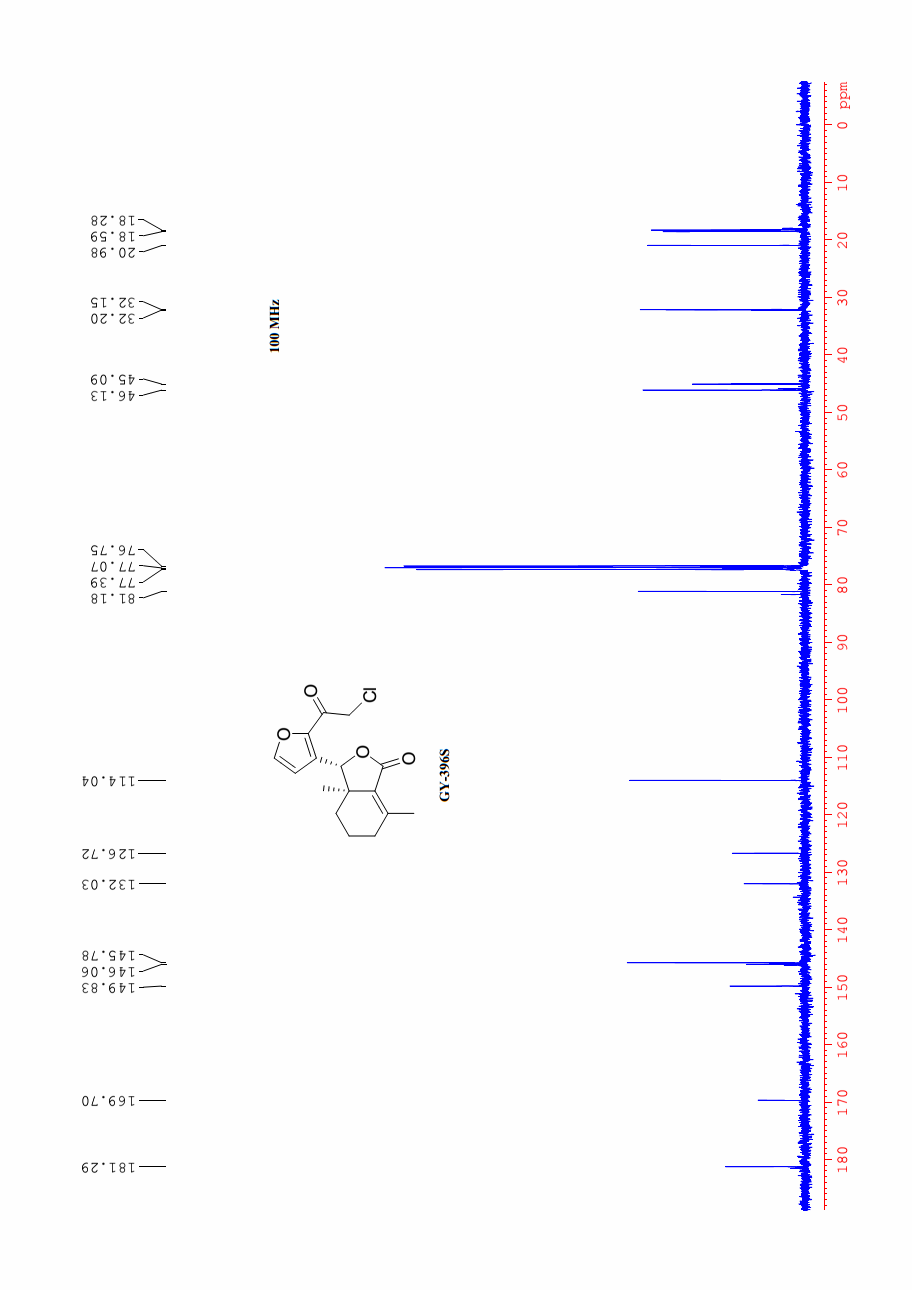


Data for **3c**:


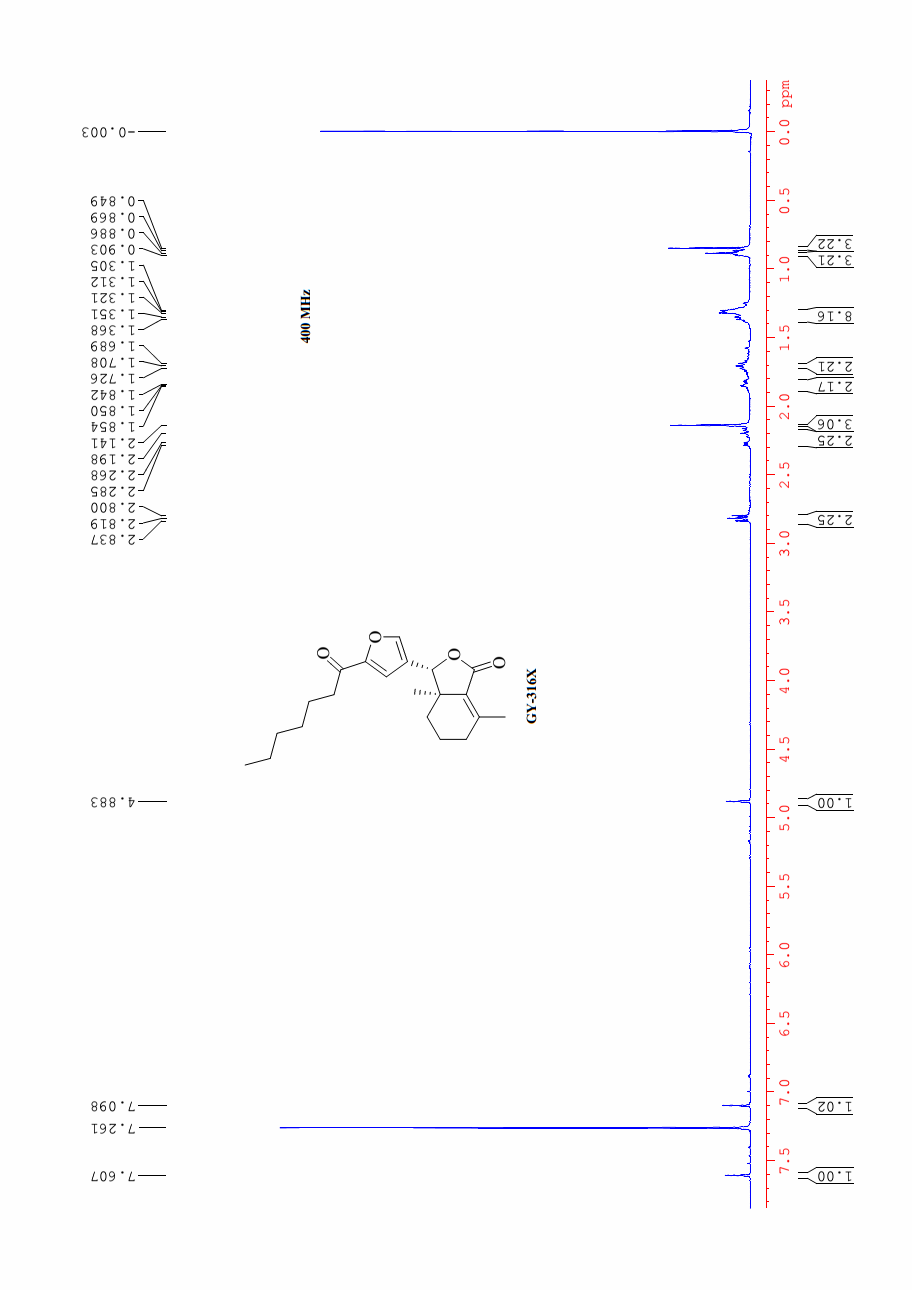

Supplement: Supplementary Information [file srep35321-s1.doc]
